# A scoping review of financial decision-making measures in midlife and beyond: results from the advancing reliable measurement in cognitive aging and decision-making ability (ARMCADA) study

**DOI:** 10.3389/fpsyg.2025.1540508

**Published:** 2025-03-17

**Authors:** Emily H. Ho, Berivan Ece, Patricia Bucko, Tatiana Karpouzian-Rogers, Sarah Pila, Zahra Hosseinian, Yasmin Hussein, S. Duke Han, Peter A. Lichtenberg, Aaron C. Lim, Sandra Weintraub, Richard C. Gershon

**Affiliations:** ^1^Department of Medical Social Sciences, Northwestern University Feinberg School of Medicine, Chicago, IL, United States; ^2^Department of Psychiatry and Behavioral Sciences, Northwestern University Feinberg School of Medicine, Chicago, IL, United States; ^3^Department of Psychology, Fordham University, New York, NY, United States; ^4^Department of Psychology, University of Southern California, Los Angeles, CA, United States; ^5^Institute of Gerontology, Wayne State University, Detroit, MI, United States; ^6^Medical and Psychological Screening Division, California Department of Human Resources, Sacramento, CA, United States; ^7^Mesulam Center for Cognitive Neurology and Alzheimer’s Disease, Northwestern University Feinberg School of Medicine, Chicago, IL, United States

**Keywords:** financial decision-making, aging, healthy aging, cognitive impairment, Alzheimer’s disease, neurocognitive disorders, dementia, financial management

## Abstract

**Background:**

Cognitive decline in older adults affects key functions such as memory, concentration, planning, reasoning, and decision-making (DM). This decline in cognitive abilities compromises basic DM skills, with growing evidence that DM can decline before noticeable impairment or an official cognitive impairment diagnosis, adversely impacting quality of life and leading to negative outcomes in financial management and daily activities.

**Objective:**

This scoping review aims to identify and evaluate existing measures of financial decision-making (FDM) abilities in clinical and community-dwelling populations aged 45 and older.

**Methods:**

We conducted a systematic search in EMBASE (Elsevier), PsycINFO, PubMed, MEDLINE, PsychARTICLES, and Web of Science for studies published between January 2018 and November 2023. The multi-domain scoping review yielded 16,278 records. Title and abstract, as well as full-text screenings, respectively, were completed by two reviewers and conflicts were resolved by PhD level researchers. We then extracted data from the full-text articles.

**Results:**

The scoping review yielded 154 articles with 96 unique measures. The most frequently used measures were variations of the Iowa Gambling Task (IGT), The Legal Capacity for Property Law Transactions Assessment Scale (LCPLTAS), the Decision-making Competence Assessment Tool (DMCAT), the temporal discounting paradigm, and the Short Form version of the Financial Capacity Instrument (FCI-SF). Commonly used measures of financial decision-making (FDM) often assessed specific aspects, such as risk-taking behavior and basic financial knowledge.

**Discussion:**

Many of the FDM measures found in this scoping review were developed for use in laboratory settings, and less is known about potential for clinical use adaptation. Future work addressing this measurement gap could significantly enhance early interventions to ameliorate or mitigate decline, thereby improving financial management and quality of life for at-risk individuals.

## 1 Introduction

Dementia in late age is commonly caused by neurodegenerative disease (Cao et al., 2020) and is characterized by cognitive decline that prevents independence in daily living activities. Dementia is a rapidly growing public health crisis, with an individual developing dementia every 3 s (Alzheimer’s Disease International, 2020)^1^. Such cognitive decline impacts everyday tasks related to financial consequences, such as managing money, paying bills, and making financial judgments (Marson et al., 2009).

Broadly defined and in line with prior operationalizations (Appelbaum et al., 2016; Gerstenecker et al., 2018; Marson et al., 2012; Widera et al., 2011), financial decision-making (FDM) is the ability to independently manage financial tasks (e.g., budgeting, investment strategies, retirement planning) by evaluating and selecting among available financial options with minimal error and/or unnecessary financial loss (Chiong et al., 2014) in a manner that aligns with personal values and self-interest.

While age alone does not determine susceptibility to fraud (Bosley et al., 2019), older adults with mild cognitive impairment (MCI) display poorer decision-making across a wide variety of contexts, including finances (Fenton et al., 2023), are more likely to fall victim to scams compared with those with normal cognition for age (Han et al., 2016), and as individuals age, they may become more susceptible to financial abuse (Federal Trade Commission, 2020^2^; Manthorpe et al., 2012; Nowrangi et al., 2022) even when cognitively healthy (Boyle et al., 2022). Chiong et al. (2014) found that financial errors, such as excessive spending or being susceptible to telephone or email scams, were common in patients with Alzheimer’s Disease and Related Dementias (ADRD). Indeed, in 2017, US financial institutions reported over 63,000 cases of elder financial exploitation, culminating in as much as 1.7 billion dollars in suspicious activity (Karp and Ortiz, 2019)^3^. Financial fraud and scams targeting older adults are prevalent, affecting roughly 1 in 18 cognitively intact older adults living in the community each year (Burnes et al., 2017).

Recent evidence suggests that financial vulnerability may serve as a precursor to incipient cognitive decline. In a large sample of Medicare beneficiaries living alone, those with a diagnosis of ADRD were more likely to miss bill payments up to 6 years before the formal diagnosis and experienced a decline in credit scores 2.5 years prior to diagnosis (Nicholas et al., 2021). Lim et al. (2024) reported that financial exploitation vulnerability was associated with worse performance in verbal memory, confrontation naming, phonemic fluency, and executive functioning in a sample of non-demented adults over 50. Furthermore, an analysis from the Health and Retirement Study (HRS) showed that compared with healthy older adults, individuals with probable dementia experienced a faster rate of decline in total household wealth (Li et al., 2023).

In concert, these recent findings suggest that symptoms of financial mismanagement and manifestations of financial vulnerability (e.g., Lichtenberg et al., 2020a,b) may percolate and manifest in the years preceding a formal ADRD diagnosis, with myriad potential adverse downstream consequences (see Chandra et al., 2023 for a recent review).

Well-validated tools are needed to assess and screen for subtle changes in FDM ability and the potential vulnerabilities that may indicate potential underlying neurodegeneration. Such tools could possibly distinguish between normal-age-related changes in FDM and changes due to neurodegenerative diseases like ADRD. By identifying such measures, it will be possible to screen for mild behavioral impairments that may precede nascent or incipient mild cognitive impairment.

However, to our knowledge, there is no synthesis of the available literature examining brief measures that assess FDM ability that may be sensitive to early changes in neurocognition. Many existing measures are typically used in legal settings when attempting to ascertain an individual’s financial capacity and competence (Ghesquiere et al., 2019), which often is enacted when impairment is already in an advanced stage, past the point where early detection measures would have clinical utility. Additionally, such measures generally require trained examiners, are lengthy (e.g., more than 1 h), and are often semi-structured interviews, which by nature limit standardization. Many cognitive screening tools also only have validation evidence in adults aged 65 and older (Anstey et al., 2019), even as there is growing consensus that risk factors for dementia emerge and accumulate throughout the life course (Livingston et al., 2024).

To address these gaps, and as a part of a larger multi-domain scoping review (Ho et al., 2024), we conducted a scoping review synthesizing the state of the literature on several fronts: (1) a survey of the FDM measures recently used in research and/or clinical settings, (2) determining the frequency of the FDM measures used, (3) ascertaining the existing psychometric evidence for FDM measures for use in early detection of cognitive decline; (4) determining the clinical populations in which such FDM measures have been validated.

## 2 Methods

This scoping review is guided by the methodological framework developed by Arksey and O’Malley (2005). The review methodology and results are reported in accordance with the PRISMA Extension for Scoping Reviews (PRISMA-ScR; Tricco et al., 2018).

### 2.1 Protocol and registration

The domain of FDM was analyzed as part of a larger scoping review. The primary aim was to identify existing decision-making measures applied to adults in midlife and later. Further protocol details can be found in the protocol of the parent multiple-domain scoping review (Ho et al., 2024). This study is exempt and classified as non-human subjects research at Northwestern University (STU00220334).

### 2.2 Search strategy and eligibility criteria

Upon establishing the research questions (Ho et al., 2024), we developed a search strategy and inclusion criteria consistent across all domains of the multi-domain scoping review. The eligibility criteria encompassed cohort studies, case–control studies, and randomized control trials that evaluated DM in adults aged 45 and above (see Supplementary Table S1 for details). Previous scoping reviews on aging and DM ability have focused on later life, typically on individuals aged 50 or 60 (e.g., Mah et al., 2021; Raj et al., 2019; Usher and Stapleton, 2022; Walsh et al., 2017). To identify tools sensitive to early changes in cognitive aging, we included articles with study populations starting from midlife (i.e., ages 45 and above). Studies on shared decision-making, decision aids, and perceptual decision-making tasks (e.g., the random dot motion task; von Lautz et al., 2019) were excluded from consideration.

We conducted a systematic search across multiple databases, including EMBASE (Elsevier), PsycINFO, PubMed, MEDLINE, PsychARTICLES, and Web of Science, to identify relevant studies published between January 1, 2018 and November 06, 2023. This timeframe was meant to capture measures currently used in clinical and research settings and those that could be easily adapted to a digital format. The scoping review used several search terms to capture three broad categories: decision-making (e.g., “decisional impairment”), financial (e.g., “financial management”), and measurement (e.g., “assessment,” “tool”). See Supplementary Table S2 for search terms specific to FDM.

### 2.3 Screening, data extraction, and synthesis

The scoping review proceeded through three phases: (1) title and abstract screening, (2) full-text review, and (3) full-text extraction and synthesis.

#### 2.3.1 Title and abstract screening

From November 10th to December 8th, 2023, a team of 18 trained reviewers conducted title and abstract screening using the online review tool Covidence (Veritas Health Innovation, 2024). Each article was independently screened by two reviewers to assess eligibility. Articles unanimously agreed upon for inclusion proceeded to the full-text review stage, while those unanimously agreed upon for exclusion were removed from further consideration. The agreement rate between reviewers was 89.5%, and disagreements (*n* = 1,705) were resolved by consultation with two expert scientists.

#### 2.3.2 Full-text review

Full-text review was conducted from December 8th to December 22nd, 2023, by 13 reviewers in Covidence (Veritas Health Innovation, 2024). Each article’s full text was independently assessed by two reviewers to confirm inclusion/exclusion using the eligibility criteria applied during title and abstract screening (see Supplementary Table S1); discrepancies were resolved by an independent third expert reviewer.

#### 2.3.3 Full-text extraction

Data extraction was conducted by eight reviewers using Qualtrics (Qualtrics ©, 2024). The following information was extracted from each article: definition of DM, assessed DM domain(s), sample characteristics (e.g., age, sample size, clinical features), and details of the measurement tools used. For each DM measure, specific details included the type of administration (e.g., in-person, remote, self-administered), required technology (e.g., computer, pen-and-paper, tablet, smartphone), and psychometric properties mentioned (e.g., reliability and validity).

#### 2.3.4 Synthesized findings

Upon completing data extraction on January 31st, 2024, all data from Qualtrics were exported to Excel for preliminary analysis. This analysis categorized all articles by relevant domains and identified measures, providing both “article-level” and “measure-level” data. Following the identification of the most frequently used measures (see Table 1; Supplementary Table S3), we further assessed their direct relevance to FDM, considering that while some measures were not frequently used, they explicitly assessed FDM. If a measure strictly focused on FDM ability, it was considered relevant (coded as 2); otherwise, measures that mentioned aspects or potential correlates of FDM (e.g., numeracy, ability to complete financial tasks such as bill payment, counting currency) were further examined (coded as 1) to determine if FDM ability was mentioned. Measures that assessed other domains (i.e., advanced care planning survey relevant to end-of-life) were removed from consideration (coded as 0).

**Table 1:** Fifteen most frequently used measures assessing FDM.

Measure	% Cited	Construct(s) assessed	Format
Iowa Gambling Task (IGT; Bechara et al., 1994)	14.3%	DM under uncertainty and risk	Computer; lab-based task
Legal Capacity for Property Law Transactions Assessment Scale (LCPLTAS; Giannouli et al., 2018)	12.3%	Basic monetary skills; cash transactions; bank statement management; bill payment; financial conceptual knowledge; FDM; knowledge of personal assets	Semi-structured interview; performance-based task
Decision Making Competence Assessment Tool (DMCAT; Finucane and Gullion, 2010) (12-item assessment)	8.4%	Comprehension; dimension weighting; cognitive reflection; consistency	Performance-based task
Temporal Discounting Task (Benzion et al., 1989; Green et al., 1994; McClure et al., 2007; McHugh and Wood, 2008; Sellitto et al., 2010)	5.2%	Impulsivity; reward valuation; time perception, self-control	Computer/questionnaire; lab-based task
Financial Capacity Instrument- Short Form (FCI-SF; Marson et al., 2016)	4.5%	Financial conceptual knowledge; monetary calculation; use of a checkbook and register; and use of a bank statement	Semi-structured interview; performance-based task
Financial Competence Assessment Inventory (FCAI; Kershaw and Webber, 2008)	4.5%	Everyday financial abilities; financial judgment; estate management; cognitive functioning related to financial tasks; debt management; support resources	Semi-structured interview; performance-based task
Lawton Instrumental Activities of Daily Living (IADL)- Finances Question (Lawton and Brody, 1969)	4.5%	Ability to handle finances	Questionnaire
Game of Dice Task (GDT; Brand et al., 2005)	3.9%	DM under risk	Computer; lab-based task
Adult- Decision Making Competence (A-DMC; Bruine de Bruin et al., 2007)	3.2%	Resistance to framing; recognizing social norms; under/over-confidence; applying decision rules; consistency in risk perception; resistance to sunk costs; path independence	Computer/pen/paper; performance-based task
Balloon Analog Risk Task (BART; Lejuez et al., 2002)	3.2%	Risk-taking	Computer; lab-based task
Financial Exploitation Vulnerability Scale (FEVS; Lichtenberg et al., 2020a,b)	2.6%	Financial situational awareness; psychological vulnerability; undue influence; past financial exploitation	Questionnaire
Lichtenberg Financial Decision Rating Scale (LFDRS; Lichtenberg et al., 2015)	2.6%	Financial situational awareness; psychological vulnerability; undue influence; past financial exploitation; intellectual factors	Semi-structured interview/questionnaire
Numerical Activities of Daily Living-Financial (NADL-F; Arcara et al., 2019)	2.6%	Counting currencies; written abilities, item purchase; percentages; financial concepts; bill payment; and financial judgments	Semi-structured interview; performance-based task
Scam Awareness Task (Boyle et al., 2019)	2.6%	Knowledge of deceptive tactics; willingness to engage in risky behaviors; perception of vulnerability	Questionnaire
Ultimatum Game (Güth et al., 1982)	2.6%	Financial altruism; fairness preferences; economic rationality	Computer; lab-based task

Column 2 indicates the percentage of the 154 articles included in the scoping review that reference each measure. For the frequency of each measure among the 227 total measures identified, refer to Supplementary Table S3.

Inter-rater agreement was evaluated based on a randomly selected subset of 23 FDM articles (15% of total articles) extracted by two independent reviewers. The inter-rater agreement was examined for all information extracted from the eligible FDM articles for both article-level and measure-level data.

## 3 Results

### 3.1 Search results: article-level

**Figure 1:**
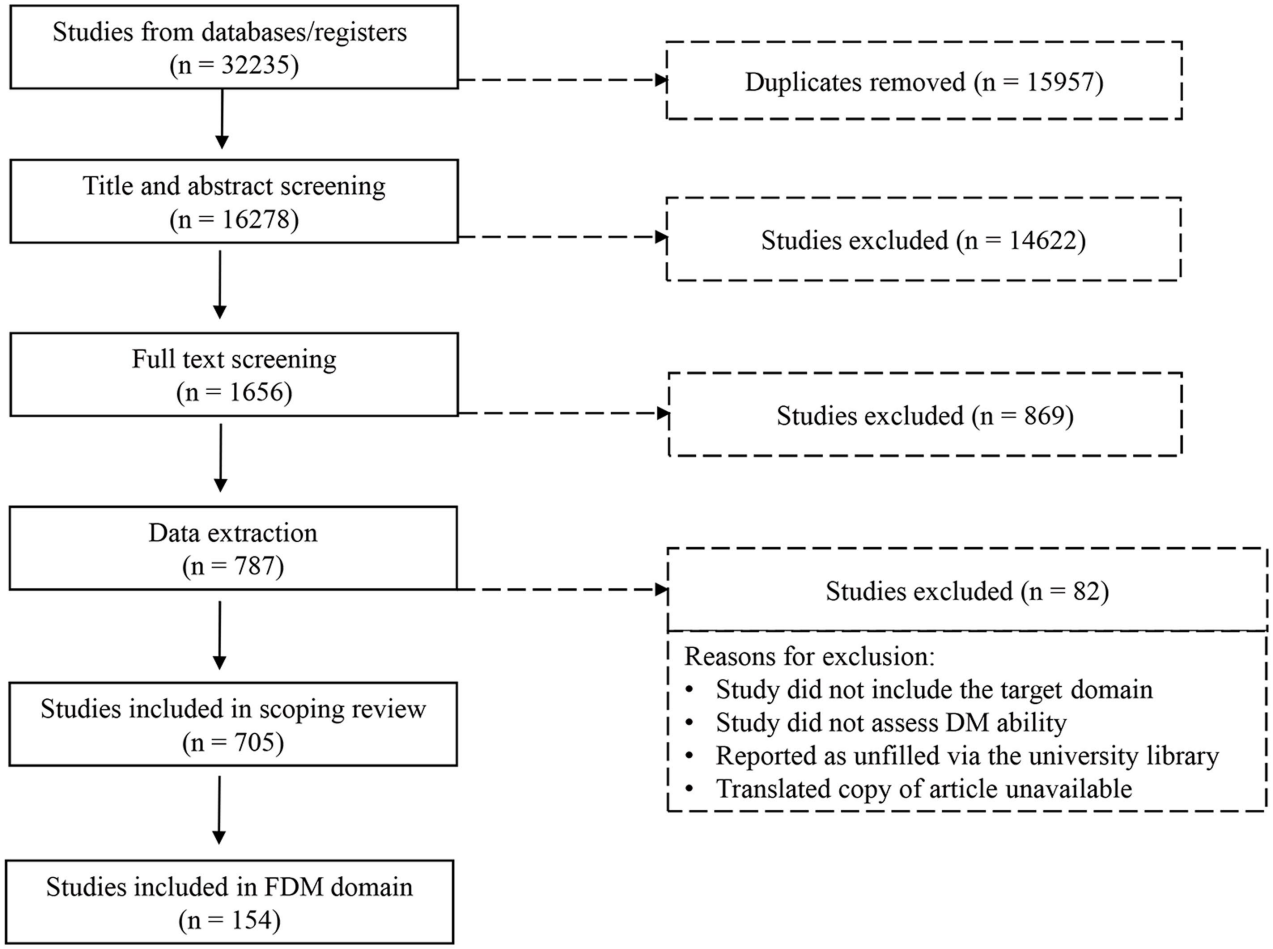
PRISMA flowchart.

The initial database search yielded 32,235 articles based on the search criteria (Figure 1). After 15,957 duplicates were removed, the remaining 16,278 articles were reviewed in Covidence (Veritas Health Innovation, 2024). The remaining articles were first screened by title and abstract, resulting in the exclusion of 14,622 articles. Following full-text screening, 869 additional articles were excluded. The remaining 787 articles advanced to the extraction phase, during which 82 more articles were excluded. Reasons for exclusion at this stage included articles that tested paradigms tangential to FDM such as reinforcement learning paradigms (Biernacki et al., 2020; Brown et al., 2020), which were determined to be unsuitable for a clinical screening context or relied only on clinician evaluation with no mention of the standardized qualitative or quantitative measure used (e.g., Gan et al., 2023). This resulted in a final set of 705 articles in the overall multi-domain scoping review. Of these 705 articles, 154 focused on FDM in adults 45 and older. Overall, 42.2% of these 154 articles focused solely on FDM, while 57.8% covered multiple domains. The full list of these 154 articles is provided in Supplementary Table S4. Finally, the PRISMA flow chart for all these stages of the current scoping review is presented in Figure 1.

### 3.2 Search results: measure-level

Among the 154 articles focusing on the FDM domain, we identified 96 unique FDM measures. A large portion (67.7%) of these 96 measures were used only once across the sample, either because they were specifically designed for the study or represented a variation of a paradigm. On the other hand, some measures were used in more than one study, resulting in the total frequency of 227 for these 96 FDM measures. For example, the Iowa Gambling Task (IGT; Bechara et al., 1994) was used in 22 studies. Frequency rate for each FDM measure is provided in Supplementary Table S5.

The five most commonly used measures to assess FDM were the Iowa Gambling Task (IGT; Bechara et al., 1994), Legal Capacity for Property Law Transactions Assessment Scale (LCPLTAS; Giannouli et al., 2018), Decision-making Competence Assessment Tool (DMCAT; Finucane and Gullion, 2010), variations of the temporal discounting task (Benzion et al., 1989; Green et al., 1994; McClure et al., 2007; McHugh and Wood, 2008; Sellitto et al., 2010), and the Financial Capacity Inventory (FCI; FCI-SF; Marson et al., 2000). These five FDM measures are briefly described below. In addition, the list of the 15 most frequent FDM measures is presented in Table 1 (see Supplementary Table S5 for the entire list of the 96 FDM measures).

- ***The Iowa Gambling Task (IGT).*** The IGT is a lab-based computerized task. In this task, participants aim to maximize their monetary accruals by selecting options with immediate large or small rewards, with a 50% probability of paying a penalty. The options containing large rewards also contain larger penalties, while those containing smaller rewards have smaller penalties. Thus, across many trials, the normative choice is to choose the options containing smaller rewards. The IGT was used a total of 22 times in the scoping review. In this scoping review, the IGT was utilized prominently in studies involving samples with psychiatric disorders and behavioral addictions (68.2%), mainly gambling addiction, and among participants with Parkinson’s disease (13.6%). Among the studies reviewed in this article, the IGT was self-administered under supervision in 63.6% and self-administered independently in 31.8%.
- ***The Legal Capacity for Property Law Transactions Assessment Scale (LCPLTAS).*** The LCPTLAS consists of seven domains, including basic monetary skills such as counting currency, cash transactions, bank statement management, bill payments, conceptual financial knowledge, financial decision-making, and asset knowledge and management (Giannouli et al., 2018). All 19 studies that used this measure in the current scoping review included participants with MCI and/or dementia. The measure was primarily administered by an examiner (84.2%), though some studies did not specify administration methods (15.8%).
- ***The Decision-making Competence Assessment Tool (DMCAT; 12-item Assessment).*** The DMCAT includes a 6-item FDM subscale, where participants are asked to select mutual funds, presented in a multiple-choice format (Bangma et al., 2021). The assessment includes three simple and three complex items that assess comprehension and integration of information provided in tables; the complex items follow the format of the simple items but present more mutual fund information and options. The task is performance-based, with a right or wrong answer for each item. All 13 studies that used this measure were longitudinal studies from the Rush Memory and Aging Project (MAP; e.g., Bennett et al., 2005; Stewart et al., 2020) in which participants were cognitively normal and dementia-free upon study entry. In most of the studies included in this review, the DMCAT was administered by an examiner (77%). The remaining studies did not report administration methods.
- ***The Temporal Discounting Task.*** Temporal discounting tasks require participants to choose between a smaller reward to be obtained immediately or a larger reward to be obtained after a specified delay. In the current sample of articles, the temporal discounting task (Benzion et al., 1989; Green et al., 1994; McClure et al., 2007; McHugh and Wood, 2008; Sellitto et al., 2010) was used eight times and primarily administered to healthy participants (50%), followed by participants with ADHD (25%), Parkinson’s disease (12.5%), and chronic acquired brain injury (12.5%). Administration methods for this measure varied; most of the studies included in the scoping review reported that this task was self-administered with no supervision and completed using a computer (62.5%), while the remaining 37.5% reported that the task was given in an interview/questionnaire-based format and administered by an examiner.
- ***The Financial Capacity Inventory- Short Form (FCI-SF).*** The FCI-SF is a performance-based measure designed to measure a broad set of financial skills and activities that allow an individual to function independently (Marson et al., 2000). Using Marson’s conceptual framework (2000) which focuses on activities of daily living, the FCI-SF assesses lower-level and higher-level performance-based activities (e.g., counting currency and counting currency and managing a checkbook, respectively) and financial judgment decisions (e.g., making investment decisions). Of the seven studies that used this measure in this scoping review, 85.7% included a clinical population with MCI and/or some form of dementia, and one (14.3%) longitudinal study included participants that were cognitively normal upon entry. All but one of the studies in this review reported that this measure was administered by an examiner (85.7%); the remaining study did not provide administration methods.

Among the DM measures identified in the current scoping review, the Lichtenberg Financial Decision Rating Scale (LFDRS; Lichtenberg et al., 2015) appeared to be the most comprehensive one, addressing DM ability alongside financial knowledge and instrumental activities of daily living (IADLs) associated with finances. It is designed in a multiple-choice format, covering key concepts essential to DM ability, such as financial situational awareness, psychological vulnerability, financial exploitation, undue influence, and current financial transactions. The LFDRS was used in 1.8% of the studies included in this scoping review. In every case, the measure was administered by an examiner. Typically, this measure was administered in-person (75%) though one article did not provide mode of administration (25%). The measure was not used in any specific clinical group, however, in all instances, it was administered to adults aged 60 and above.

### 3.3 Measure characteristics

We extracted information for all unique FDM measures identified on their (1) modality of administration (i.e., remote vs. in-person), (2) self-administration and supervision, and (3) the technology (e.g., computer, tablet) used, (4) duration of administration, and (5) psychometric evidence (i.e., reliability and validity).

*Modality of administration.* Most of the measures (74%) were conducted in-person. Remote and hybrid administration of the measures was less common; remote administration accounted for 15.6% of the total measures and hybrid administration accounted for 5.2% of the total measures. Modality of administration was not specified for the remaining 5.2% of the measures.

*Examiner or self-administration.* Approximately a quarter (25.1%) were self-administered under supervision, and 26.4% were reported as self-administered with no examiner supervision. More than a third (38.3%) of the measures were administered by an examiner or clinician. For the remaining 10.1% of measures, no information was reported on self-administration and supervision.

*Technology format.* Nearly half of the measures (47.6%) required some form of technology, most commonly a computer. Of 227 applications of 96 DM measures reviewed in this article, 8.8% were administered via computer and remotely. The remaining studies either used paper/pen (5.3%) or did not report any use of technology (47.1%).

*Duration of administration.* Most articles did not report on the measures’ duration to completion (91.2%) and psychometric properties of internal consistency (87.6%), test–retest reliability (98.5%), and inter-rater reliability (96.4%).

### 3.4 Sample characteristics

**Figure 2:**
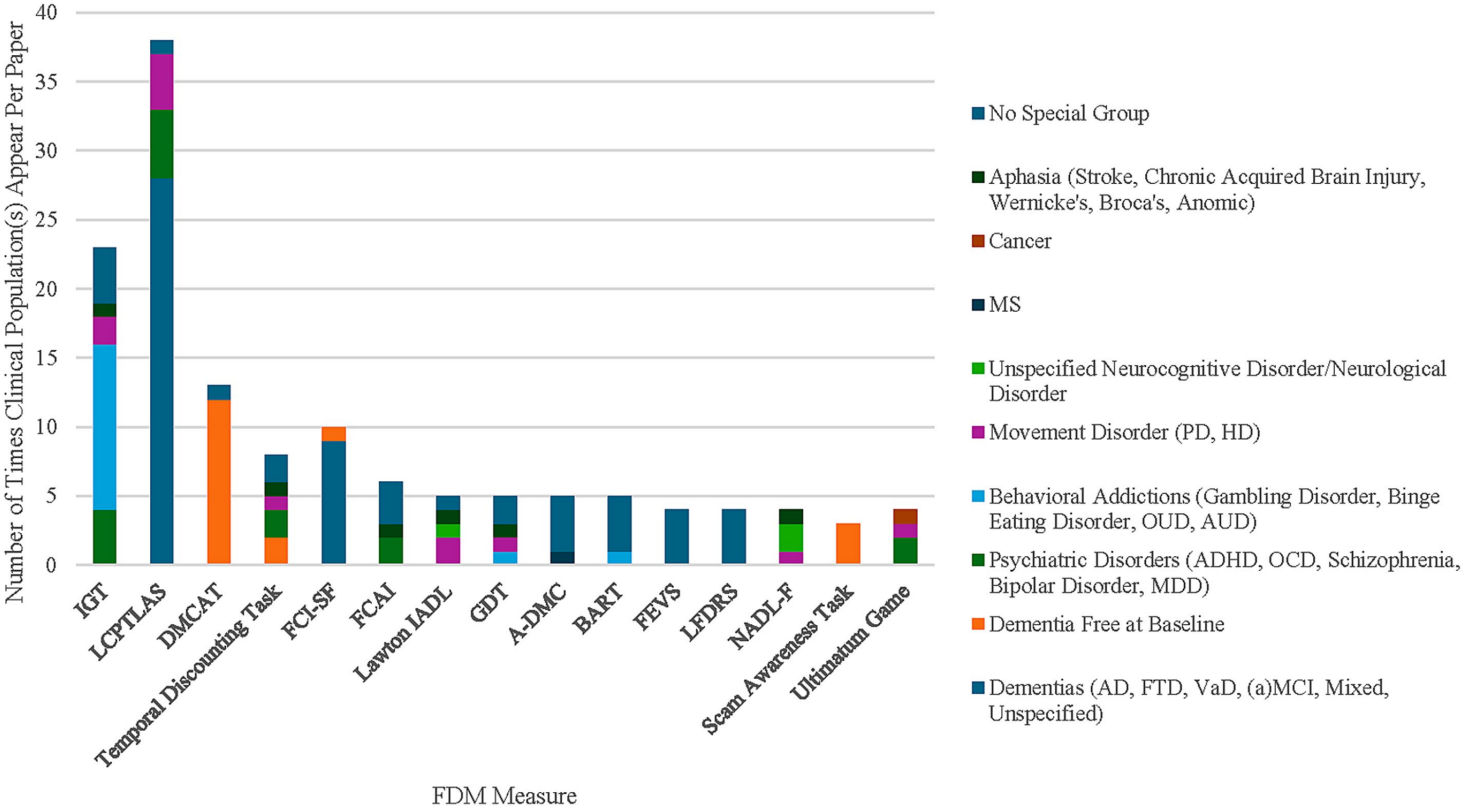
Stacked bar chart depicting the number of times clinical population appears in articles for 15 of the most used measures. (1) IGT = Iowa Gambling Task; LCPTLAS = Legal Capacity for Property Law Transactions Assessment Scale; DMCAT = Decision-making Competence Assessment Tool; FCI-SF = Financial Capacity Instrument- Short Form; FCAI = Financial Competence Assessment Inventory; Lawton IADL = Lawton Instrumental Activities of Daily Living; GDT = Game of Dice Task; A-DMC = Adult Decision-making Competence Scale; BART = Balloon Analog Risk Task; FEVS = Financial Exploitation Vulnerability Scale; LFDRS = Lichtenberg Financial Decision Rating Scale; NADL-F = Numerical Activities of Daily Living- Financial. (2) CI = Cognitive Impairment; Movement Disorders included PD = Parkinson’s Disease and HD = Huntington’s Disease; MS = Multiple Sclerosis; Cancer included prostate cancer specifically; VaD = Vascular dementia; FTD = Frontotemporal dementia; AD = Alzheimer’s Disease; MCI = Mild Cognitive Impairment; aMCI = amnestic Mild Cognitive Impairment; Behavioral Addictions included GD = Gambling Disorder, BSD = Binge Spectrum Disorder, OUD = Opioid Use Disorder, AUD = Alcohol Use Disorder, and Cannabis Use Disorder; Psychiatric Disorders included ADHD = Attention Deficit Hyperactivity Disorder, OCD = Obsessive Compulsive Disorder, Schizophrenia, BPD=Bipolar Disorder, MDD = Major Depressive Disorder. (3) Some articles included multiple clinical populations (e.g., though the IGT was the most frequently used measure, it included fewer clinical populations across all studies than the LCPTLAS and Lawton IADL). (4) Dementia free at baseline categorizes longitudinal aging studies (e.g., Rush Memory and Aging) in which all participants were free of dementia during their baseline assessment.

Studies included in the present scoping review were examined regarding their sample characteristics (e.g., age range, diagnosis, and language). Among all reviewed studies, 93.5% explicitly reported that they involved participants aged 45 and older, while the remaining 6.5% could have been inferred for older populations based on their clinical diagnosis (e.g., mild cognitive impairment (MCI); dementia of the AD type (predominantly amnestic); posterior cortical atrophy, a syndrome in which visuospatial decline precedes other types of cognitive impairment) which typically affects populations over the age of 45. Across all studies, 70.8% included participants over the age of 65. With respect to the inclusion of clinical samples, 40.9% of 154 studies did not include a clinical group, 40.9% included both clinical samples and healthy controls, and 18.2% included only clinical samples (Figure 2). Of the 154 studies included in this scoping review, 13% included multiple clinical groups (e.g., participants with schizophrenia and Huntington’s disease). The most common clinical populations were patients with a diagnosis of mild cognitive impairment (MCI) due to Alzheimer’s Disease (AD) as well as other forms of dementia (e.g., frontotemporal, vascular) in 24% of the studies. Additionally, 21.4% of the studies included samples with psychiatric disorders (e.g., bipolar disorder, schizophrenia, major depressive disorder, obsessive-compulsive disorder, and various behavioral addictions and substance use disorders). Movement disorders, including Parkinson’s disease (PD), Huntington’s disease (HD), and Amyotrophic lateral sclerosis (ALS) were mentioned in 7.8% of the articles. Neurologic conditions (e.g., stroke, multiple sclerosis, brain lesions) were mentioned in 7.1% and various health conditions (e.g., HIV, cancer, frailty) were mentioned in 1.9% of articles. Finally, results regarding the language of administration in the study demonstrated that approximately half of the studies were conducted in English (53.9%), while the rest were conducted in other languages, such as Greek (13%), Italian (8.4%), German (6.5%), Dutch (2.6%), and Japanese (3.2%).

## 4 Discussion

The purpose of this scoping review was to synthesize the state of the literature for measures of FDM in mid-life adults to older populations. We identified 96 unique measures from 154 articles that met the inclusion criteria. As noted in other work (Marroni et al., 2017; Sudo and Laks, 2017), the ability to manage finances is mediated by various other abilities, including cognitive function and numeracy. This review revealed a substantial number of studies focusing on FDM, as differentiated from measures specifically designed to assess FDM from other cognitive abilities (e.g., executive functions, attention, and memory).

FDM measures identified in this scoping review appear to be broadly grouped into four categories: (1) lab-based tasks (e.g., IGT and temporal discounting task), (2) tasks specifically assessing financial knowledge and abilities (e.g., FCI-SF and LCPTLAS), (3) financial performance tasks (e.g., DMCAT), and (4) self-reported financial knowledge and FDM ability tasks [e.g., LFDRS and Financial Exploitation Vulnerability Scale (FEVS; Lichtenberg et al., 2020a,b; see Table 1; Supplementary Table S3)]. However, it should be noted that these categories are not mutually exclusive. For example, the FCI-SF and LCPTLAS both include financial performance tests (e.g., writing a check correctly and counting currency) and assessment of self-reported financial knowledge and abilities (e.g., knowledge of assets and making investment decisions).

All existing DM measures have their own strengths and weaknesses. For example, lab-based tasks [e.g., IGT, Game of Dice Task (GDT; Brand et al., 2005), Ultimatum Game (UG; Güth et al., 1982)] were frequently used in the studies, but they are not easily adaptable for clinical use and seem to be more relevant for assessing impulsivity. Measures such as the FCI-SF, Financial Competence Assessment Inventory (FCAI; Kershaw and Webber, 2008), and Numerical Activities of Daily Living-Financial (NADL-F; Arcara et al., 2019) are related more to executive functioning and financial knowledge rather than the ability to evaluate and select financial options effectively. Other measures, such as the DMCAT, are not self-report but rather performance-based, and presuppose a high level of extant financial literacy, such as using highly specialized terms (e.g., mutual funds) that can pose a problem for effectively assessing abilities if high financial literacy is a necessary precondition.

On the other hand, some other measures such as the LFDRS (Lichtenberg et al., 2015) includes a conceptual model that assesses FDM through the interplay of intellectual factors, contextual elements, and an individual’s value system, making them valuable tools for evaluating FDM ability in both research and practical settings.

In this review, FDM is defined as the ability to independently manage tasks like budgeting, investing, and retirement planning with minimum error or financial loss (Chiong et al., 2014). The results of our scoping review, however, revealed a wide heterogeneity of operationalizations for FDM. Specifically, the term “FDM” is often used broadly, encompassing financial knowledge and daily financial tasks rather than focusing on the ability to make financial decisions. Additionally, many existing measures emphasize factors that influence FDM, such as knowledge, task execution, risk-taking, and impulsivity, without directly assessing FDM ability itself. Moreover, commonly used measures are often lab-based tasks, limiting scalability for more widespread screening or continued monitoring in a clinical context.

Despite challenges related to scalability and assessment of peripheral factors and outcomes of FDM (e.g., risk-taking, impulsivity, executive functions) in some existing measures, it is important to acknowledge that performance on such measures can give valuable insights into real-world financial decision-making. Meier and Sprenger (2010) found associations in performance in the temporal discounting paradigm task with individuals’ credit reports, suggesting that impulsive decision-makers were more likely to have more credit card debt. Similarly, while financial exploitation does not encompass financial decision-making ability, the FEVS has effectively differentiated between older adults who have and have not fallen victim to fraud (Lichtenberg et al., 2021). Such evidence emphasizes that FDM measures may reveal how FDM evolves in cognitively impaired populations and how such shifts may impact daily financial choices and activities.

The current review has many strengths. By focusing on measures tailored to assess FDM, our review provides valuable insights into tools that may detect early and subtle changes in FDM abilities. Our review employed a minimum age of 45 years, with the goal of detecting early FDM difficulties before potential clinical manifestations of cognitive impairment. Understanding these measures’ use in midlife has the potential to contribute to timely intervention and better patient outcomes. Additionally, the current review included scales administered in any language to ensure a broad and inclusive review of available DM measures; however, by restricting the publication language to English (the language of the authors) we may have inadvertently excluded relevant articles in other languages. Further, the exclusion of gray literature (unpublished dissertations, theses, white papers, etc.) may have introduced a publication bias and left out potentially relevant articles and measures. Including only peer-reviewed published articles, however, ensured the inclusion of measures that have undergone formal scientific review process and have been repeatedly tested in multiple studies. An additional limitation is that the vast majority of studies did not report validity or reliability estimates, and the study samples’ race/ethnicity (e.g., White) were relatively homogenous. The inclusion of such information can enhance generalizability and improve ecological validity, or the extent to which the scientific findings or measures can be applied to real-world populations. Given that dementia is a condition that disproportionately affects Latinx and Black populations (e.g., Quiñones et al., 2020; Quiroz et al., 2022), future studies should focus on increasing ethnoracial representativeness in their study samples (see Glover et al., 2024 for a recently proposed model to increase community engagement and research participation among diverse older adults).

### 4.1 Future directions

Many older adults with cognitive impairment—as high as 7.4 million older adults in the US, according to one study (Li et al., 2022)—are managing their household finances, increasing the likelihood of adverse financial consequences or mismanagement. Nicholas et al. (2021) showed, in a large cohort study of more than 80 thousand Medicare beneficiaries living alone, that those with ADRD were more likely to miss bill payments up to 6 years prior to a formal clinician diagnosis, and Lichtenberg et al. (2024) show that earlier memory loss predicts excess spending. These recent findings suggest there are potentially highly detectable indicators, even while an individual presents as cognitively normal. Furthermore, those unaware of their cognitive decline are more likely to suffer financial loss (Mazzonna and Peracchi, 2024), highlighting the gains that can be achieved with earlier detection.

This scoping review identified numerous measures for detecting and characterizing aspects of financial decision-making decline. Future research should aim to build upon these existing tools so they can be implemented as individuals transition from middle to late adulthood, potentially making early identification of FDM deficits a possibility. Earlier detection of financial vulnerability may allow for earlier entry points for individuals to be targeted for a variety of interventions, such as those related directly to potential dementia diagnosis and treatment, as well as functional collateral consequences. For example, evidence of early potential financial mismanagement may prompt clinicians or family members to seek a more thorough neuropsychological evaluation for the individual. Recently tested interventions, such as the randomized clinical trial Plan Your Lifespan, showed that a knowledge-based, interactive intervention increased understanding, accessing, and planning for health decisions for aging in place in cognitively healthy adults aged 65 and above (Lindquist et al., 2017). Early planning, for example, of intra-family-member transfers of FDM responsibilities, can help reduce burden on the family and potentially on public resources. Early detection of signs of financial vulnerability with standardized screening measures may serve as a timely prompt for a variety of decisions related to household finance planning, ultimately preserving financial independence and autonomy for as long as possible.

There is also a growing call to better understand, characterize, and sensitively measure the behavioral and social risk factors that predict ADRD manifestation (Harrell et al., 2024; Martínez-Nicolás et al., 2021). Advances in health measurement in this area include, for example, the development and validation of smartphone-based ecological momentary testing paradigms such as the NIA-funded Mobile Toolbox project (Gershon et al., 2022; Landavazo et al., 2023; Rentz et al., 2024), passive digital signatures gleaned from sources like electronic data warehouses and medical records (e.g., Boustani et al., 2020) or remote home or individual sensing technologies such as accelerometry through real-world navigation tasks.

There may also be additional diagnostic value from information procured from caregivers, whether they be from standardized questionnaires (Olde Rikkert et al., 2011) or new technologies and assessment paradigms that have a growing evidence-base in dementia patients, and could easily be applied to caregivers, such as in-home sensors. For example, a study looking at remote sensors in homes of dementia patients (Gaugler et al., 2019) suggests that movement patterns in the home may be able to detect early impairment.

Finally, these measurement development and validation efforts should be informed by specific individual circumstances, such as extant household assets (Lee et al., 2024), norms surrounding intra-household exchange and sharing of financial resources (Bertocchi et al., 2014), and other social determinants of health and cultural norms and values regarding how financial decisions are made. Ultimately, personalized approaches using a combination of theoretically and data-guided indices may enhance measurement precision, greatly aiding in mitigating the severe economic burdens of age-related neurodegenerative disease.

## 5 Conclusion

Recognizing the consequences of cognitive aging on financial decision-making (FDM) and its broader implications for society is critical for developing effective strategies and solutions to address the challenges posed by an aging population. Systematic and well-validated tools are needed to identify and detect such vulnerabilities in FDM for several reasons. Earlier detection of FDM vulnerabilities can promote financial agency and security, preserve autonomy, and guide discussions around life-planning and surrogate decision-making (Hsu and Willis, 2013). While financial decision-making measures from middle to late adulthood can potentially detect subtle changes in FDM, the scoping review identified that the five most used measures in literature from the past 5 years may be difficult to implement in a systematic and comprehensive ways. The current review mainly identified lab-based decision-making measures, measures that require a relatively high degree of financial literacy, and self-report questionnaires that can be inherently biased. While the measures provide insights into everyday financial decision-making, there are challenges in applying outcomes of these measures to FDM tendencies of the general adult population. Although this scoping review identified some gaps in the FDM measures that are actively being used in recent years, these measures provide important insights into various aspects of financial decision-making. Future research and clinical initiatives should aim to implement this information in a more systematic, validated, and broadly applicable manner.

## Data Availability

The original contributions presented in the study are included in the article/Supplementary material, further inquiries can be directed to the corresponding author.

## Footnotes

^1^ https://www.alzint.org/about/dementia-facts-figures/dementia-statistics/

^2^ https://www.ftc.gov/system/files/documents/reports/consumer-sentinel-network-data-book-2020/csn_annual_data_book_2020.pdf

^3^ https://www.consumerfinance.gov/about-us/blog/financial-institutions-report-widespread-elder-financial-abuse/
