# Supplementary material for: A scoping review of financial decision-making measures in midlife and beyond: results from the advancing reliable measurement in cognitive aging and decision-making ability (ARMCADA) study
Source: Front Psychol. 2025 Mar 17;16:1540508. doi: 10.3389/fpsyg.2025.1540508 (PMC11955626; doi:10.3389/fpsyg.2025.1540508)
Supplement: Supplementary file 1 [file Data_Sheet_1.docx]

Supplementary Materials

Index

| **Supplementary Material** | **Page(s)** |
| --- | --- |
| Table S1: Inclusion and exclusion criteria | 2 |
| Table S2: Financial decision-making search terms | 3 |
| Table S3: Fifteen most frequently used financial decision-making measures | 4-10 |
| Table S4: Article-level information of articles included in the scoping review | 10-16 |
| Table S5: Measure-level information of unique financial decision-making measures in the scoping review | 16-20 |
| Table S6: PRISMA-ScR Checklist | 20-23 |
| References (Studies cited in Table S3) | 24-26 |

# Table S1. Inclusion and exclusion criteria for articles (Ho et al., 2024).

|  | Inclusion criteria | Exclusion criteria |
| --- | --- | --- |
| Population | At least one group of individuals over 45 years | Adults ≤ 45 years old |
| Study Characteristics | The study mentions at least one assessment of a DM ability in a target domain  The domain of interest is an outcome assessed by the study | Single-subject - case studies  Focus groups  Review articles  Narrative reviews  Grey literature (e.g. conference proceedings, dissertations)  Books, book chapters  Commentaries  Other non-research publications |
| Other | Language: All languages as long as manuscript is in English  Location: All geographical locations | Articles that only measure shared decision-making  Articles that only measure decision aids  Perceptual/Low-level decision making |

# Table S2. Search terms specific to the financial decision-making domain.

| Search Terms |
| --- |
| ' economic decision making'  'fraud'  'financial abuse'  'financial literacy'  'financial management'  'personal finance'  'household income'  'economic decision*'  'force choice  'forced choice'  'game theory paradigm'  'monetary decision*'  'risky decision*  'finance'  ‘finances’  ‘financial’  ‘economic’  ‘money’  ‘monetary’  ‘scam’  ‘scams’ |

**3** **Table S3.** Fifteen most frequently used measures assessing FDM.

| **Measure** | **Measure Description** | **Measure Frequency** | **Citing Articles** | **Clinical Groups** | **Age Range** | **Psychometric Properties** | **Format** | **In-person vs. Remote** | **Examiner vs. Self-administered** |
| --- | --- | --- | --- | --- | --- | --- | --- | --- | --- |
| Iowa Gambling Task (IGT; Bechara et al., 1994) | Participant chooses cards from four decks with an endowment of fake money; two of the decks are advantageous and two are disadvantageous. Players are instructed to select one card at a time and try to lose the least amount of money. | 22 (9.7%) | Aksu et al., 2023; Bailis et al., 2021; Balconi et al., 2018; Bangma et al., 2019; Ghosh et al., 2020; Giustiniani et al., 2019; Grassi et al., 2020; Kim et al., 2020; Koerts et al., 2021; Mallorqui-Bague et al., 2018; Moccia et al., 2021; Munguia et al., 2021; Nigro et al., 2018; Orduz-Bastidas et al., 2020; Pettorruso et al., 2019; Ramchandran et al., 2020; Rooks et al., 2020; Schellenberg & Bailis, 2018; Tannou et al., 2021; Weller et al., 2019; Wilson & Vassileva, 2018; Wolfling et al., 2020 | Attention Deficit Hyperactivity Disorder (ADHD); Obsessive Compulsive Disorder (OCD); Problem gamblers/ Gambling Disorder (GD); Parkinson’s Disease (PD); Binge Spectrum Disorder (BSD); Abstinent substance users; Patients with aphasia (Wernicke’s/Broca’s/Anomic); Opioid Use Disorder (OUD); Substance dependence (opioids, marijuana) | 18-95 | Not reported | Computer; Lab-based task | In-person | Self-administered no supervision;  Self-administered under supervision |
| Legal Capacity for Property Law Transactions Assessment Scale (LCPLTAS; Giannouli et al., 2018) | Using the theoretical model developed by the FCI, the LCPLTAS assesses financial knowledge and skills that are adapted for the Greek population. It includes 7 domains: basic monetary skills, cash transactions, bill payment, bank statement management, financial knowledge, FDM, and awareness of personal assets. | 19(8.4%) | Giannouli et al., 2018; Giannouli et al., 2019^a^; Giannouli & Tsolaki, 2019^b^; Giannouli & Tsolaki, 2021^a^; Giannouli & Tsolaki, 2021^b^; Giannouli & Tsolaki, 2021^c^; Giannouli & Tsolaki, 2021^d^; Giannouli & Tsolaki, 2021^e^; Giannouli & Tsolaki, 2022^a^; Giannouli & Tsolaki, 2022^b^; Giannouli & Tsolaki, 2022^c^; Giannouli & Tsolaki, 2022^d^; Giannouli & Tsolaki, 2022^e^;  Giannouli et al., 2022^f^; Giannouli & Tsolaki, 2022^g^; Giannouli & Tsolaki, 2023^a^; Giannouli & Tsolaki, 2023^b^; Giannouli & Tsolaki, 2023^c^; Stamovlasis et al., 2022; | Alzheimer’s Disease (AD); Mild Cognitive Impairment (MCI); Unspecified dementias; Frontotemporal Dementia (FTD); Parkinson’s Disease (PD); Multiple Sclerosis (MS); Huntington’s Disease (HD); Mixed dementia with depression; Unspecified neurocognitive disorders; Vascular Dementia (VaD) | 44-98 | Healthy participants Cronbach’s alpha= 0.93  Clinical population Cronbach’s alpha=0.75  Test-retest Pearson’s r=0.97, p<0.001  (Giannouli et al., 2018) | Semi-structured interview guide; Performance-based task | In-person | Administered by examiner |
| Decision Making Competence Assessment Tool (DMCAT; Finucane & Gullion, 2010) (12-item assessment) | Performance task consisting of 12 items (6 financial and 6 healthcare). The FDM subscale consists of 6 items where mutual funds are selected based on predetermined preferences. The total score is derived from the number of correct responses, with higher scores reflecting better FDM. | 13 (5.7%) | Glover et al., 2021; Han et al., 2020; Kapasi et al., 2021; Lamar et al., 2020; Stewart et al., 2019; Stewart et al., 2020; Stewart et al., 2022; Sunderaraman et al., 2020; Weissberger et al., 2021; Wilson et al., 2023; Yu et al., 2021; Yu et al., 2021; Yu et al., 2022 | Alzheimer’s Disease(AD)*; Various dementias*; Unspecified chronic conditions*; Parkinson’s Disease (PD)*; Motor impairment*; Physical frailty and disability* | 30-103 | Cronbach’s alpha=0.75  (Stewart et al., 2020) | Performance-based task | In-person | Administered by examiner |
| Temporal Discounting Task  (Benzion et al., 1989; Green et al., 1994; McClure et al.,2007; McHugh & Wood, 2008; Selitto et al., 2010) | A temporal discounting task evaluates how people value rewards at different times. Participants are asked to choose between smaller, immediate rewards and larger, delayed rewards. This task helps determine the extent to which individuals devalue future rewards compared to immediate ones, shedding light on their time-related preferences and decision-making processes. It is often used in research on impulsivity, delay discounting, and self-control. | 8 (3.5%) | Aiello et al., 2019; Bangma et al., 2019; Koerts et al., 2021; Lockenhoff & Samanez-Larkin, 2019; Seaman et al., 2018; Sunderaraman et al., 2019; Wilson et al., 2023; Yu et al., 2021 | Attention Deficit Hyperactivity Disorder (ADHD); Parkinson’s Disease (PD); Chronic acquired brain injury | 18-100 | Not reported | Computer/Questionnaire; Lab-based task | Hybrid | Administered by examiner;  Self-administered no supervision |
| Financial Capacity Instrument- Short Form. (FCI-SF; Marson e al., 2016) | The Financial Capacity Instrument–Short Form (FCI-SF; Tolbert et al., 2019) evaluates advanced financial skills and is a streamlined version of the FCI. It covers five domains: (1) ‘mental calculation,’ (2) ‘financial conceptual knowledge,’ (3) ‘single checkbook/register task,’ (4) ‘complex checkbook/register task,’ and (5) ‘using bank statements.’ | 7 (3.1%) | Gonzalez et al., 2021; Mimmack et al., 2023; Nowrangi et al., 2022; Steward et al., 2019; Steward et al., 2019; Tolbert et al., 2019; Vassilaki et al., 2022 | Alzheimer’s Disease (AD); Mild Cognitive Impairment (MCI); Frontotemporal Dementia (FTD); Parkinson’s Disease (PD); Multiple Sclerosis (MS); Huntington’s Disease (HD) | 44-95 | Cronbach’s alpha=0.90 (Mimmack et al., 2023) | Semi-structured interview guide; Performance-based task | In-person | Administered by examiner |
| Financial Competence Assessment Inventory (FCAI; Kershaw & Webber, 2008) | A 38-item psychological assessment administered individually to objectively measure six aspects of financial competence: everyday financial skills, financial judgment, estate management, cognitive functions related to financial tasks, debt management, and the use of support resources. | 7 (3.1%) | Bangma et al., 2019; Coundouris et al., 2023; Gill et al., 2019; Koerts et al., 2021; Sunderaraman et al., 2019; Sunderaraman et al., 2022; Sunderaraman et al., 2022 | Alzheimer’s Disease (AD); Mild Cognitive Impairment (MCI; Frontotemporal Dementia (FTD); Attention Deficit Hyperactivity Disorder (ADHD); Chronic acquired brain injury | 18-85+ | Cronbach’s alpha (total)= > 0.80 for all subscales except “financial resources” (Cronbach’s alpha= 0.54)  (Koerts et al., 2019) | Semi-structured interview guide; Performance-based task | In-person | Administered by examiner |
| Lawton Instrumental Activities of Daily Living (IADL; Lawton & Brody, 1969)- Finances Question | Tool designed to evaluate a person's ability to carry out more complex daily tasks beyond basic self-care activities. The finances question pertains to handling finances (managing money, paying bills, and budgeting). | 7 (3.1%) | Arcara et al., 2019; Burgio et al., 2022; Geng et al., 2020; Giannouli et al., 2018; Gill et al., 2019; Hall et al., 2023; Sousa et al., 2021; | Alzheimer’s Disease (AD); Mild Cognitive Impairment (MCI; Frontotemporal Dementia (FTD); Unspecified neurological disorders/ Major and minor neurocognitive disorders | 45-98 | Not reported | Questionnaire | In-person | Administered by examiner |
| Game of Dice Task (GDT; Brand et al., 2005) | Task evaluates how executive functions impact decision-making in gambling scenarios. Participants aim to increase their starting capital over 18 dice rolls by choosing either a single number or a combination of two, three, or four numbers. After each roll, results and gains or losses are displayed along with an acoustic signal indicating success. Choices of single or two-number combinations are considered disadvantageous (lower winning probability but higher potential losses), while three or four-number combinations are advantageous (higher winning probability with lower losses). | 6 (2.6%) | Aksu et al., 2023; D'Aurizio et al., 2019; Kim et al., 2021; Mueller & Brand, 2018; Mueller & Brand, 2018; Tannou et al., 2021 | Premanifest Huntington’s disease (HD); Aphasia (Anomic/Wernicke's/Broca's) | 18-95 | Not reported | Computer; Lab-based task | In-person | Self-administered no supervision;    Self-administered under supervision |
| Adult- Decision Making Competence (A-DMC; Bruine de Bruin et al., 2007) | 87-item test designed to assess how well individuals make decisions. Includes 7 subscales: resistance to framing, recognizing social norms, under/overconfidence, applying decision rules, consistency in risk-perception, resistance to sunk costs, and path independence. | 5 (2.2%) | Del Missier et al., 2020; Eberhardt et al., 2018; Hoffmann et al., 2020; Mueller et al., 2020; Sobkow et al., 2020 | Multiple sclerosis (MS) | 18-88 | Cronbach’s alpha= 0.79 (A-DMC modified only resistance to framing) (Mueller et al., 2020)  Cronbach’s alpha subscales (modified German version) =  0.59 (Resistance to sunk costs)  0.72 (Resistance to framing)  0.56 (Under/over confidence)  0.68 (Applying decision rules)  0.59 (Consistency in risk perception)  (Hoffmann et al., 2020) | Computer/Pen/paper; Performance-based task | Hybrid | Self-administered no supervision |
| Balloon Analog Risk Task (BART; Lejuez et al., 2002) | Designed to assess risk preferences in a choice scenario in which participants inflate a computerized balloon with the goal of inflating it to maximum capacity but not letting it pop. With each successful inflation, participants are rewarded 5 cents. If the balloon pops, participants lose all their earned money. | 5 (2.2%) | Aksu et al., 2023; Quan et al., 2022; Rooks et al., 2020; Sebri et al., 2023; Tannou et al., 2021 | Opioid Use Disorder (OUD) | 18-95 | Not reported | Computer; Lab-based task | In-person | Self-administered no supervision;  Self-administered under supervision |
| Financial Exploitation Vulnerability Scale (FEVS; Lichtenberg et al., 2020) | 17-item self-report scale on financial, psychological, and relationship insecurities around personal finance. Used to identify victims of financial exploitation. | 4 (1.8%) | Aksu et al., 2023; Hall et al., 2022; Lichtenberg et al., 2020; Lichtenberg et al., 2021 | N/A | 45-85+ | Cronbach’s alpha= 0.78  (Hall et al., 2022) | Questionnaire | Hybrid | Self-administered no supervision |
| Lichtenberg Financial Decision Rating Scale (LFDRS; Lichtenberg et al., 2015) | 77 multiple-choice questions divided into subscales that assess Financial Situational Awareness, Psychological Vulnerability, Susceptibility to Undue Influence, Past Financial Exploitation, and Intellectual Factors. It functions as a rating scale to evaluate these dimensions. | 4 (1.8%) | Flores & Lichtenberg et al, 2023; Lichtenberg et al., 2018; Lichtenberg et al., 2020; Rooks et al., 2020 | N/A | 45-85+ | Cronbach’s alpha=0.71  (Rooks et al., 2019)  Cronbach’s alpha for subscales=  0.75 (Financial situational awareness)  0.72 (Psychological Vulnerability)  0.7 (Susceptibility to undue influence)  0.7 (Susceptibility to financial exploitation)  (Lichtenberg et al.,2018) | Semi-structured interview guide;  Questionnaire | In-person | Administered by examiner |
| Numerical Activities of Daily Living-Financial (NADL-F; Arcara et al., 2019) | A battery designed to evaluate capabilities related to managing money transactions in daily life, including handling family and personal finances, and making financial decisions. The NADL-F is organized into seven domains, each assessing performance in real-world scenarios and addressing activities that likely engage multiple cognitive functions. | 4 (1.8%) | Arcara et al., 2019; Benavides-Varela et al., 2020; Burgio et al., 2022; Sousa et al., 2021 | Major and minor neurocognitive disorders; Mild Cognitive Impairment (MCI); Cerebral stroke; Parkinson’s Disease (PD); Unspecified neurological disorders | 44-89 | Cronbach’s alpha (overall)= 0.81  (Sousa et al., 2022)  Cronbach’s alpha subscales=  0.62 (counting currencies)  0.66 (reading abilities)  0.77 (item purchase)  0.74 (percentages)  0.64 (financial concepts)  0.65 (bill payments)  0.61 (financial judgment)  Interrater Reliability ICC (subscales)=  1 (counting currencies)  1 (reading abilities)  0.98 (item purchase)  1 (percentages)  0.96 (financial concepts)  0.92 (bill payments)  0.95 (financial judgments)  (Arcara et al., 2019) | Semi-structured interview guide; Performance-based task | In-person | Administered by examiner |
| Scam Awareness Task (Boyle et al., 2019) | A 7-point Likert scale featuring 5 statements is used to evaluate individuals' receptiveness to sales pitches (items 1, 2, and 5), their interest in potentially risky investments (item 3), and their awareness of vulnerability related to age (item 4). | 4 (1.8%) | Kapasi et al., 2021; Wilson et al., 2023; Yu et al., 2021; Yu et al., 2021 | Alzheimer’s Disease(AD)*; Various dementias*; Unspecified chronic conditions*; Parkinson’s Disease (PD)*; Motor impairment*; Physical frailty and disability* | 58-103 | Cronbach’s alpha=0.54 (Yu et al, 2021) | Questionnaire | In-person | Administered by examiner |
| Ultimatum Game (Güth et al., 1982) | A 'proposer' is given a certain amount of money and decides how much of it to share with a 'responder'. The responder then chooses whether to accept the offer, in which case both players receive the proposed amount, or reject it, in which case neither player receives any money. | 4 (1.8%) | Brune et al., 2020; Holtfrerich et al., 2020; Mukherjee et al., 2020; Persson et al., 2019 | Prostate cancer; Major Depressive Disorder (MDD); Huntington’s Disease (HD); Schizophrenia | 18-84 | Not reported | Computer; Lab-based task | Hybrid | Self-administered under supervision;  Self-administered no supervision |

*Note. (1) Measure frequency, shown in the third column, indicates how often a measure was mentioned among the 227 total measures identified in the scoping review. For percentages reflecting the proportion of the 154 papers that cite each measure, see Table 1 in the manuscript. (2) Psychometric properties were not reported for most measures. While certain measures appeared across multiple articles, very few articles mentioned internal consistency, test-retest reliability, and inter-rater reliability. (3) * Indicates clinical populations in longitudinal studies from the Memory and Aging Project at Rush University (Kapasi et al., 2021; Wilson et al., 2023; Yu et al., 2021; Yu et al., 2021); participants were free of such conditions at baseline.*

**4** **Table S4.** Article level information of the 154 papers included in the financial domain.

| **Author(s)** | **Language(s)** | **Sample Age** | **Clinical Groups** |
| --- | --- | --- | --- |
| Adamkovic, 2020 | Other- not indicated | 18-64 | No special group |
| Ahmed et al., 2020 | English | Not provided | Posterior cortical atrophy/ AD |
| Aiello et al., 2019 | Other- not indicated | 45-84 | PD |
| Aksu et al., 2023 | Other- not indicated | 18-64 | Opioid Use Disorder (OUD) |
| Anderson et al, 2021 | English | 18-64 | Bipolar disorder/ Healthy controls |
| Arcara et al, 2019 | Italian | 45-84 | Neurological disorders/ Healthy controls |
| Asaoka et al, 2020 | Japanese | 18-72 | Behavioral addictions/Healthy controls |
| Bailis et al., 2021 | English | 18-85 | Problem Gamblers/ Casino Patrons |
| Balconi et al, 2018 | Italian | 45-75 | PD |
| Bangma et al., 2019 | German | 19-64 | Adults w/ ADHD/ Healthy controls |
| Banks et al., 2019 | English | 47-50 | No special group |
| Benavides-Varela et al., 2020 | Italian | 45-85+ | MCI/ Healthy controls |
| Berezuk et al., 2018 | English | 45-85+ | MCI |
| Bosley et al., 2019 | English | 18-84 | No special group |
| Brune et al., 2020 | German | 18-64 | HD/ Schizophrenia/ Healthy controls |
| Burgio et al., 2022 | Italian | 56-85 | MCI |
| Campbell & Lichtenberg, 2020 | English | 45-84 | No special group |
| Charness et al., 2020 | Other- not indicated | 18-92 | No special group |
| Chen et al., 2018 | English | 18-85+ | No special group |
| Coundouris et al., 2023 | English | 60-88 | No special group |
| Danesin et al., 2022 | English | 45-85+ | MCI/ PD/ Left or right hemisphere stroke/ Healthy controls |
| D'Aurizio et al., 2019 | Italian | 22-60 | Pre-manifest HD/ Healthy controls |
| Del Missier et al., 2020 | Swedish | 60-85 | No special group |
| DeLiema, 2018 | English | 65-85+ | Participants who experienced elder mistreatment |
| Eberhardt et al., 2018 | English | 18-88 | No special group |
| Fareri et al., 2022 | English | 63-80 | No special group |
| Fenton et al., 2023 | English | Not provided | MCI |
| Flores & Lichtenberg, 2023 | English | 60-85 | No special group |
| Fujino et al., 2018 | Japanese | 18-64 | Gambling Disorder/ Healthy controls |
| Geng et al., 2020 | Chinese | 45-85+ | AD/ MCI/ Healthy controls |
| Gerrans et al., 2021 | English | Not provided | No special group |
| Gerstenecker et al., 2018 | English | 50-91 | AD |
| Gerstenecker et al., 2019 | English | 45-95+ | MCI/Advanced cognitive impairment/ Healthy controls |
| Gerstenecker et al., 2022 | English | 57-91 | AD/ Healthy controls |
| Ghosh et al., 2020 | English/ Other- not indicated | 18-55 | Opioid Dependence/ Cannabis Dependence/ Healthy controls |
| Giannouli & Tsolaki, 2019 | Greek | 58-91 | PD/PD+Depression/Depression/Healthy controls |
| Giannouli & Tsolaki, 2021 | Greek | 45-85+ | Mild AD (APOE e4 allele carriers)/ AD (non-carriers)/ Healthy controls |
| Giannouli & Tsolaki, 2021 | Greek | Not provided | AD/ Healthy controls |
| Giannouli & Tsolaki, 2021 | Greek | 45-84 | MCI |
| Giannouli & Tsolaki, 2021 | Greek | 45-84 | Vascular dementia w/ depression/ Vascular dementia w/o depression/ Depression w/o VD/ Healthy controls |
| Giannouli & Tsolaki, 2022 | Greek | 65-84 | Mild AD |
| Giannouli & Tsolaki, 2022 | Greek | 45-85+ | FTD/ Healthy controls |
| Giannouli & Tsolaki, 2022 | Greek | 45-84 | No special group |
| Giannouli & Tsolaki, 2022 | Greek | 54-89 | Single domain aMCI/ Multiple domain aMCI/ Healthy controls |
| Giannouli & Tsolaki, 2022 | Greek | 65-89 | Mild AD/ Single and multiple domain aMCI/ Healthy controls |
| Giannouli & Tsolaki, 2022 | Greek | 65-84 | Mild AD |
| Giannouli & Tsolaki, 2023 | Greek | 47-98 | Dementia/Dementia and depression/ Depression/ Healthy controls |
| Giannouli & Tsolaki, 2023 | Greek | 65-84 | Mild AD and aMCI |
| Giannouli & Tsolaki, 2023 | Greek | 65-98 | AD/ Health controls |
| Giannouli & Tsolaki, 2019 | Greek | 45-85+ | MCI |
| Giannouli & Tsolaki, 2021 | Greek | 57-91 | PD/ FTD/ Healthy controls |
| Giannouli & Tsolaki, 2022 | Greek | 65-85+ | Neurocognitive disorders/ Healthy controls |
| Giannouli et al., 2018 | Greek | 45-98 | Dementia/ Healthy controls |
| Giannouli et al., 2022 | Greek | 54-98 | Amnestic MCI/ Healthy controls |
| Gill et al., 2019 | English | 65-85+ | AD/ FTD/ pheno/poss bvFTD/ Healthy controls |
| Giustiniani et al., 2019 | French | 21-59 | No special group |
| Glover et al., 2021 | English | 58-100 | No special group |
| Gonzalez et al., 2021 | English | 56-95 | Amnestic MCI/ AD/ Healthy controls |
| Grassi et al., 2020 | Italian | 18-65 | OCD and GD |
| Grimm et al., 2021 | Dutch/ German | 18-64 | ADHD/ ADHD + Comorbid Disorders/ Healthy controls |
| Hall et al., 2021 | English | 18-55 | HIV positive/ Healthy controls |
| Hall et al., 2022 | English | 45-84 | No special group |
| Hall et al., 2023 | English | 55-85+ | No special group |
| Han et al., 2020 | English | 65-85+ | No special group |
| Hasuzawa et al., 2022 | English | 18-84 | OCD/ Healthy controls |
| Hoffmann et al., 2020 | German | 19-76 | MS/ Healthy controls |
| Holtfrerich et al., 2020 | German | 50-79 | Prostate cancer (treatment and no treatment)/ Healthy controls |
| Hoven et al., 2023 | Dutch | 18-64 | Gambling Disorder/ Healthy controls |
| Irmen et al., 2018 | German | 52-75 | Idiopathic PD (w/DBS)/ Healthy controls |
| Jabraelli et al., 2021 | Persian | 18-64 | No special group |
| Kapasi et al., 2021 | English | 71-103 | AD (pathologic and nonpathologic)/ Healthy controls |
| Kim et al., 2021 | English | 40-75 | Aphasia (Anomic/Wernicke's/ Broca's)/ Healthy controls |
| Kobayashi et al., 2019 | Japanese | 45-84 | PD w/ impulse control disorder/ PD w/o ICD/ Healthy controls |
| Koerts et al., 2021 | German | 18-64 | ADHD/ Healthy controls |
| Lamar et al., 2020 | English | 58-100 | No special group |
| Lenglin et al., 2023 | English | 45-84 | bvFTD/ Adult onset brain lesions/ Healthy controls |
| Li et al., 2021 | English | 18-86 | No special group |
| Lichtenberg et al., 2018 | English | 60-85+ | No special group |
| Lichtenberg et al., 2020 | English | 60-85+ | No special group |
| Lichtenberg et al., 2020 | English | 60-85+ | No special group |
| Lichtenberg et al., 2020 | English | 45-84 | No special group |
| Lichtenberg et al., 2021 | English | 60-85+ | No special group |
| Lichtenberg et al., 2021 | English | Not provided | No special group |
| Lichtenberg et al., 2023 | English | 65-84 | No special group |
| Lighthall et al., 2018 | English | 18-84 | No special group |
| Limbrick-Oldfield et al., 2020 | English | 20-58 | Gambling Disorder/ Healthy controls |
| Lockenhoff & Samanez-Larkin, 2019 | English | 18-95 | No special group |
| Lohse et al., 2023 | Danish | 19-52 | No special group |
| Mallorqui-Bague et al., 2018 | Spanish | 18-65 | Gambling disorder |
| Martin et al., 2019 | English | Not provided | MCI/ Mild AD/ Healthy controls |
| Martin-Fernandez et al., 2018 | Dutch | <18-84 | No special group |
| Mimmack et al., 2023 | English | Not provided | MCI/ Healthy controls |
| Moccia et al., 2021 | Italian | 18-65 | Gambling Disorder/ Healthy controls |
| Morin et al., 2019 | English | 65-85+ | Depression/ Healthy controls |
| Muda et al., 2018 | Polish | 18-64 | No special group |
| Mueller & Brand, 2018 | German | 18-62 | No special group |
| Mueller et al., 2018 | German | 18-68 | No special group |
| Mueller et al., 2020 | English | 21-82 | No special group |
| Mukherjee et al., 2020 | English | 18-84 | MDD/ Healthy controls |
| Munguia et al., 2021 | Spanish | 18-64 | Binge spectrum disorder/ Binge spectrum disorder w/ compulsive buying/ Healthy controls |
| Nigro et al., 2018 | Italian | 18-70 | Problem Gamblers |
| Nowrangi et al., 2022 | English | 65-85+ | MCI/ Healthy controls |
| O'Brien & Hess, 2020 | English | 18-89 | No special group |
| Ojala et al., 2018 | Dutch | 18-52 | Gambling Disorder/ Healthy controls |
| Orduz-Bastidas et al., 2020 | Spanish | 45-84 | PD/ Healthy controls |
| Perez et al., 2018 | English | 28-79 | No special group |
| Persson et al., 2019 | Swedish | 27-58 | No special group |
| Peters et al., 2020 | English | 20-47 | Gamblers/ Healthy controls |
| Pettorruso et al., 2019 | Italian | 18-65 | Gambling disorder |
| Phan et al., 2023 | Vietnamese | 18-65 | No special group |
| Pornapattananangkul et al., 2018 | Other- not indicated | 45-84 | No special group |
| Quan et al., 2022 | English | 21-50 | No special group |
| Ramchandran et al., 2020 | English | 55-89 | No special group |
| Reeck et al., 2022 | English | 18-64 | No special group |
| Rooks et al., 2020 | English | 45-84 | No special group |
| Santos et al., 2022 | Portugese | 45-85+ | Mild to moderate AD |
| Schellenberg & Bailis, 2018 | English | 18-85 | Casino Patrons (potential problem gamblers) |
| Seaman et al., 2018 | English | 22-83 | No special group |
| Sebri et al., 2023 | Italian | 18-64 | No special group |
| Sharman et al., 2019 | English | 18-84 | Gambling Disorder |
| Sobkow et al., 2020 | Polish | 18-55 | No special group |
| Sousa et al., 2021 | Portugese | 52-89 | Mild neurocognitive disorders/ Major neurocognitive disorders/ Healthy controls |
| Spaniol et al., 2018 | Italian | 33-75 | vmPFC damage/ Healthy controls |
| Spataro & Bella, 2021 | Italian | 54-68 | ALS/ Healthy controls |
| Stamovlasis et al., 2022 | Greek | 45-98 | AD/ Cognitive impairment/ Healthy controls |
| Steward et al., 2019 | English | 54-88 | MCI-ASD/ MCI-AMD/ Healthy controls |
| Steward et al., 2019 | English | 54-88 | MCI |
| Stewart et al., 2019 | English | 65-85+ | No special group |
| Stewart et al., 2020 | English | 58-100 | No special group |
| Stewart et al., 2022 | English | 45-85+ | No special group |
| Sugden et al., 2019 | English | 18-55 | No special group |
| Sullivan et al., 2022 | English | 18-77 | No special group |
| Sunderaraman et al., 2019 | English | 30-81 | Chronic acquired brain injury/ Healthy controls |
| Sunderaraman et al., 2020 | English | 30-84 | No special group |
| Sunderaraman et al., 2022 | English | 57-80 | No special group |
| Sunderaraman et al., 2022 | English | 57-84 | No special group |
| Suzuki, 2018 | Japanese | 19-79 | No special group |
| Takeuchi et al., 2020 | Japanese | 18-54 | Gambling disorder/ Healthy controls |
| Tannou et al., 2021 | English | 58-95 | No special group (4 studies) /PD/ Healthy controls (1 study) |
| Thompson et al., 2021 | English | 22-72 | No special group |
| Toffano et al., 2021 | Italian | 45-85+ | Neurological disorders/ Healthy controls |
| Tolbert et al., 2019 | English | 55-90 | MCI/ AD/ Healthy controls |
| Vassilaki et al., 2022 | English | 70-85+ | No special group |
| Weissberger et al., 2021 | English | 45-85+ | No special group |
| Weller et al., 2019 | English | 24-84 | No special group |
| Wild et al., 2022 | English | 70-85+ | No special group |
| Wilson & Vassileva, 2018 | Bulgarian | 18-50 | Abstinent substance users/ Healthy controls |
| Wilson et al., 2023 | English | 58-100 | No special group |
| Wisner et al., 2021 | English | 18-46 | Schizophrenia/ Schizoaffective disorder/ Healthy controls |
| Wolfling et al., 2020 | German | Not provided | Gambling disorder and internet gaming disorder |
| Wu et al., 2018 | English | 18-70 | ASD/ Healthy controls |
| Yu et al., 2021 | English | 59-102 | Chronic conditions (AD, Dementias, PD, frailty/disability) |
| Yu et al., 2021 | English | 62-95 | No special group |
| Yu et al., 2022 | English | 59-100 | No special group |

**5** **Table S5.** Measure level information for 96 unique FDM measures (227 measures in total).

| **Measure** | **Original Citation of Measure** | **In-person vs. Remote** | **Administration** | **Technology** |
| --- | --- | --- | --- | --- |
| Adult Decision-making Competence Scale (A-DMC) (n=5) | Bruine de Bruin et al., 2007 | Hybrid | Self-administered with no supervision | Computer; Paper/pen |
| Airplane Game | Neuffer, 1987 | In-person | Administered by examiner/researcher/clinician | Paper/pen |
| Allais Paradox Lottery Task | Not provided | Remote | Self-administered with no supervision | Computer |
| Ambiguity Tolerance Task (Mukherjee et al., 2020) | Not provided | In-person | Self-administered under supervision | Computer |
| Balloon Analogue Risk Task (BART) (n=5) | Lejuez et al., 2002 | In-person | Self-administered with no supervision; Self-administered under supervision | Computer |
| Beads Task | Garety et al., 1991 | In-person | Self-administered under supervision | Computer |
| Cambridge Gambling Task (CGT) (n=3) | Rogers et al., 1999 | In-person | Self-administered under supervision | Computer |
| Choice Task- Monetary | Lenglin et al, 2023 | In-person | Self-administered under supervision | Computer |
| Columbia Card Task (CCT) (n=2) | Figner & Voelki, 2004 | In-person | Self-administered under supervision | Computer |
| Competence in Decision Rules (CDR) (n=2) | Bangma et al., 2017 | In-person | Self-administered with no supervision | Computer |
| Credit Card Repayment Decision Task | Amar et al., 2011 | Remote | Self-administered with no supervision | Computer |
| Current Financial Activities Report (CFAR) | Not provided | In-person | Not provided | Not provided |
| Decsion-making Quality Index- Financial Competence | Ambuehl et al., 2014 | Remote | Self-administered with no supervision | Computer |
| Decision-making Competence Assessment Tool (DMCAT) (n=13) | Finucane & Gullion, 2010 | In-person | Administered by examiner/researcher/clinician | Not provided |
| Delay Discounting Task (DDT) (Mukherjee et al., 2020) | Not provided | In-person | Self-administered under supervision | Computer |
| Dictator Game (n=2) | Forsythe et al., 1994 | Hybrid | Self-administered with no supervision | Computer |
| Die Roll Task (Irmen et al., 2019) | Not provided | In-person | Self-administered under supervision | Computer |
| Domain-Specific Risk-Taking Scale (DOSPERT) (n=2) | Blais & Weber, 2006 | In-person | Self-administered with no supervision; Self-administered under supervision | Computer |
| Drift Diffusion Model (DDM) Risk-taking Task | Ratcliff et al., 2016 | In-person | Self-administered under supervision | Computer |
| Economic Choice Task | Not provided | In-person | Self-administered under supervision | Computer |
| Economic Decision-making Questionnaire | Tversky & Kahneman, 1981 | In-person | Administered by examiner/researcher/clinician | Paper/pen |
| Emotional Arousal and Decision Strategies Questionnaire | Not provided | In-person | Self-administered under supervision | Computer |
| Estate Planning Documents Questionnaire (EPDQ) | Sullivan et al., 2022 | In-person | Self-administered with no supervision | Computer |
| Failure to Minimize Portfolio Risk | Banks et al., 2018 | Remote | Self-administered with no supervision | Computer |
| Financial and Health Literacy Scale | Not provided | In-person | Administered by examiner/researcher/clinician | Not provided |
| Financial Assessment and Capacity Test (FACT) | Black et al., 2007 | In-person | Administered by examiner/researcher/clinician | Not provided |
| Financial Awareness Scale | Sunderaraman et al., 2020 | In-person | Administered by examiner/researcher/clinician | Not provided |
| Financial Capacity Instrument (FCI) (n=4) | Marson et al., 2000 | In-person | Administered by examiner/researcher/clinician | Not provided |
| Financial Capacity Instrument-Short Form (FCI-SF) (n=7) | Marson et al., 2000; Gerstenecker et al., 2016 | In-person | Administered by examiner/researcher/clinician | Not provided |
| Financial Competence Assessment Inventory (FCAI) (n=7) | Kershaw & Webber, 2008 | In-person | Administered by examiner/researcher/clinician | Not provided |
| Financial Decision Tracker (FDT) (n=2) | Lichtenberg et al., 2015 | Hybrid | Administered by examiner/researcher/clinician | Computer |
| Financial Decision-making Behavior Task | Kershaw & Webber, 2008 | In-person | Self-administered with no supervision | Not provided |
| Financial Decision-Making Interview (FDMI) (n=2) | Bangma et al., 2017 | In-person | Administered by examiner/researcher/clinician | Not provided |
| Financial Exploitation Vulnerability Scale (FEVS) (n=4) | Lichtenberg et al., 2020 | In-person | Self-administered with no supervision | Paper/Pen |
| Financial Exploitation Vulnerability Scale-Short Form (FEVS-SF) | Lichtenberg et al., 2020 | In-person | Self-administered with no supervision | Not provided |
| Financial Judgment Scale | Earl et al., 2015 | Remote | Self-administered with no supervision | Computer |
| Financial Risky Choice Paradigm | De Martino et al., 2006 | In-person | Self-administered under supervision | Computer |
| fMRI Slot Machine Task | Not provided | In-person | Self-administered under supervision | Computer |
| Functional Assessment Questionnaire (FAQ) (n=2) | Pfeffer et al., 1982 | In-person | Administered by examiner/researcher/clinician | Not provided |
| Gambling Task (O'Brien & Hess, 2020) | O'Brien & Hess, 2020 | In-person | Self-administered under supervision | Not provided |
| Gambling Task (Ojala et al., 2018) | Abdellaoui et al., 2008 | In-person | Self-administered under supervision | Computer |
| Game of Dice Task (GDT) (n=6) | Brand et al., 2005 | In-person | Self-administered with no supervision; Self-administered under supervision | Computer |
| German Socio-Economic Panel Study Task | Von Rosenbladt & Stocker, 2005 | Remote | Self-administered with no supervision | Not provided |
| Holt-Laury Risky Choice Test | Holt & Laury, 2002 | In-person | Self-administered under supervision | Paper/pen |
| Incentive-Compatible Risk Preference Task | Huettel et al., 2006 | In-person | Self-administered under supervision | Computer |
| Independent Living Scale (ILS) (n=4) | Loeb, 1996 | In-person | Administered by examiner/researcher/clinician | Telephone |
| Insurance Task | Charness et al., 2020 | Not provided | Not provided | Computer |
| Intertemporal Choice Task (Geng et al., 2020) | Figner et al., 2010 | In-person | Self-administered under supervision | Computer |
| Intertemporal Choice Task (Li et al., 2021) | Toubia et al., 2013 | Remote | Self-administered with no supervision | Computer |
| Investment Game (Suzuki, 2018) | Not provided | In-person | Self-administered under supervision | Computer |
| Iowa Gambling Task (IGT) (n=22) | Bechara et al., 1994 | In-person | Self-administered with no supervision; Self-administered under supervision | Computer |
| Item Response Task | Bartels et al., 2021 | Remote | Self-administered with no supervision | Computer |
| Legal Capacity for Property Law Transactions Assessment Scale (LCPLTAS) (n=19) | Giannouli et al., 2018 | In-person | Administered by examiner/researcher/clinician | Not provided |
| Legal Capacity for Property Law Transactions Assessment Scale- Short Form (LCPLTAS-SF) (n=6) | Giannouli et al., 2018 | In-person | Administered by examiner/researcher/clinician | Not provided |
| Lichtenberg Financial Decision-Making Rating Scale (LFDRS) (n=4) | Lichtenberg et al., 2015 | In-person | Administered by examiner/researcher/clinician | Not provided |
| Lichtenberg Financial Decision-Making Rating Scale- Short Form (LFDRS-SF) | Lichtenberg et al., 2015 | Not provided | Administered by examiner/researcher/clinician | Telephone |
| Lottery Game Task | Dohmen et al., 2011 | Remote | Self-administered with no supervision | Not provided |
| Lottery Task (Anderson et al., 2021) | Fehr et al., 2007 | Remote | Self-administered with no supervision | Not provided |
| Lottery Task (Phan et al., 2023) | Not provided | In-person | Administered by examiner/researcher/clinician | Not provided |
| Lottery Task (Sobkow et al., 2020) | Cokely & Kelley, 2009 | Remote | Self-administered with no supervision | Computer |
| Lottery Task (Sugden et al., 2019) | Not provided | In-person | Self-administered under supervision | Computer |
| Minnesota Trust Game (MTG) | Johnson et al., 2009 | In-person | Self-administered under supervision | Computer |
| Mixed-Gambles Task (Hoven et al., 2023) | Hoven et al., 2023 | In-person | Self-administered under supervision | Computer |
| Monetary Choice Questionnaire (MCQ) (n=3) | Kirby et al., 1999 | Hybrid | Self-administered with no supervision; Self-administered under supervision | Computer; Paper/pen |
| Monetary Incentive Delay (MID) Task | Not provided | In-person | Self-administered under supervision | Computer |
| Money Management Self-Report Scale | Garðarsdóttir & Dittmar, 2012 | Remote | Self-administered with no supervision | Computer |
| Money Prediction Question (Mukherjee et al., 2020) | Not provided | In-person | Self-administered under supervision | Computer |
| Mortgage Task | Charness et al., 2020 | Not provided | Not provided | Computer |
| Numerical Activities of Daily Living–Financial (NADL-F) (n=4) | Arcara et al., 2017 | In-person | Administered by examiner/researcher/clinician | Paper/pen |
| The Numerical Activities of Daily Living- Financial (NADL-F) Short Version (n=2) | Arcara et al, 2017 | In-person | Administered by examiner/researcher/clinician | Not provided |
| "Pig" Dice Game | Scarne, 1945; Meder et al., 2016 | In-person | Self-administered under supervision | Computer |
| Portfolio Task | Charness et al., 2020 | Not provided | Not provided | Computer |
| Price Search Task (Sugden et al., 2019) | Not provided | In-person | Self-administered under supervision | Computer |
| Probabilistic Discounting Task | Levy & Glimcher, 2011 | In-person | Self-administered under supervision | Computer |
| Risk Approximation Task (RAT) | Dietrich et al., 2015 | In-person | Self-administered under supervision | Computer |
| Risk Aversion Task (n=2) | Barsky et al., 1997; Glenn et al.,2008; Harrison et al., 2007 | In-person | Administered by examiner/researcher/clinician | Not provided |
| Risk Choice Experiment Survey (25-items) | Carvalho & Silverman, 2017 | Remote | Self-administered with no supervision | Computer |
| Risk Tolerance Task (Mukherjee et al., 2020) | Not provided | In-person | Not provided | Not provided |
| Risky Choice Task | Not provided | In-person | Self-administered under supervision | Computer |
| Scam Awareness Task (n=4) | Boyle et al., 2019 | In-person | Administered by examiner/researcher/clinician | Paper/pen |
| Sequence-Construction Task- modified | Loewenstein & Prelec, 1993 | Not provided | Self-administered with no supervision | Computer |
| Sokol-Hessner's Mixed Gambles Task | Sokol-Hessner et al., 2009 | In-person | Self-administered with no supervision | Computer |
| Staircase Model Risk Preference Loss Task | Falk et al., 2016 | Remote | Self-administered with no supervision | Computer |
| Staircase Model Risk Preference Reward Task | Falk et al., 2016 | Remote | Self-administered with no supervision | Computer |
| Sunk Cost Task | Fujino et al., 2016 | In-person | Self-administered with no supervision | Computer |
| Temporal Discounting Task (n=8) | Benzion et al., 1989 | In-person | Administered by examiner/researcher/clinician; Self-administered with no supervision | Computer |
| The Cups Task (n=2) | Weller et al., 2007 | In-person | Self-administered with no supervision; Self-administered under supervision | Computer |
| The Lawton Instrumental Activities of Daily Living (IADL) Scale (Finances Question) (n=7) | Lawton & Brody, 1968 | In-person | Administered by examiner/researcher/clinician | Paper/pen |
| The Past and Present Financial Capacity Form (PPFCF) | Wadley et al., 2003 | In-person | Self-administered under supervision | Computer |
| The Testamentary Capacity Instrument (TCI) | Walsh et al., 1997 | In-person | Administered by examiner/researcher/clinician | Not provided |
| Timed Instrumental Activities of Daily Living (TIADL) (Finances Subscale) (n=2) | Owsley et al., 2001 | In-person | Administered by examiner/researcher/clinician; Self-administered under supervision | Computer |
| Tom's Mixed Gambles Task | Tom et al., 2007 | In-person | Self-administered with no supervision | Computer |
| Trust Game (n=2) | Berg et al., 1995 | In-person | Self-administered with no supervision | Computer |
| Ultimatum Game (n=4) | Guth et al., 1982 | Hybrid | Self-administered under supervision; Self-administered with no supervision | Computer |
| Willingness-to-Pay (WTP) Task | Becker et al., 1964 | In-person | Administered by examiner/researcher/clinician | Not provided |
| Willingness-to-Wait (WTW) Task (Mukherjee et al., 2020) | Not provided | In-person | Self-administered under supervision | Computer |

**6** **Table S6.** PRISMA ScR Checklist.

Preferred Reporting Items for Systematic reviews and Meta-Analyses extension for Scoping Reviews (PRISMA-ScR) Checklist

| **SECTION** | **ITEM** | **PRISMA-ScR CHECKLIST ITEM** | **REPORTED ON PAGE #** |
| --- | --- | --- | --- |
| **TITLE** | | | |
| Title | 1 | Identify the report as a scoping review. | 1 |
| **ABSTRACT** | | | |
| Structured summary | 2 | Provide a structured summary that includes (as applicable): background, objectives, eligibility criteria, sources of evidence, charting methods, results, and conclusions that relate to the review questions and objectives. | 2 |
| **INTRODUCTION** | | | |
| Rationale | 3 | Describe the rationale for the review in the context of what is already known. Explain why the review questions/objectives lend themselves to a scoping review approach. | 2-3 |
| Objectives | 4 | Provide an explicit statement of the questions and objectives being addressed with reference to their key elements (e.g., population or participants, concepts, and context) or other relevant key elements used to conceptualize the review questions and/or objectives. | 3 |
| **METHODS** | | | |
| Protocol and registration | 5 | Indicate whether a review protocol exists; state if and where it can be accessed (e.g., a Web address); and if available, provide registration information, including the registration number. | 4 |
| Eligibility criteria | 6 | Specify characteristics of the sources of evidence used as eligibility criteria (e.g., years considered, language, and publication status), and provide a rationale. | 4 and Table S1 and S2 |
| Information sources* | 7 | Describe all information sources in the search (e.g., databases with dates of coverage and contact with authors to identify additional sources), as well as the date the most recent search was executed. | 4 and Ho et al.. 2024 (see references) |
| Search | 8 | Present the full electronic search strategy for at least 1 database, including any limits used, such that it could be repeated. | Ho et al., 2024 |
| Selection of sources of evidence† | 9 | State the process for selecting sources of evidence (i.e., screening and eligibility) included in the scoping review. | 4-5 |
| Data charting process‡ | 10 | Describe the methods of charting data from the included sources of evidence (e.g., calibrated forms or forms that have been tested by the team before their use, and whether data charting was done independently or in duplicate) and any processes for obtaining and confirming data from investigators. | 4-5 |
| Data items | 11 | List and define all variables for which data were sought and any assumptions and simplifications made. | 5 |
| Critical appraisal of individual sources of evidence§ | 12 | If done, provide a rationale for conducting a critical appraisal of included sources of evidence; describe the methods used and how this information was used in any data synthesis (if appropriate). | N/A |
| Synthesis of results | 13 | Describe the methods of handling and summarizing the data that were charted. | 5 |
| **RESULTS** | | | |
| Selection of sources of evidence | 14 | Give numbers of sources of evidence screened, assessed for eligibility, and included in the review, with reasons for exclusions at each stage, ideally using a flow diagram. | 5-6 |
| Characteristics of sources of evidence | 15 | For each source of evidence, present characteristics for which data were charted and provide the citations. | Table S4 |
| Critical appraisal within sources of evidence | 16 | If done, present data on critical appraisal of included sources of evidence (see item 12). | N/A |
| Results of individual sources of evidence | 17 | For each included source of evidence, present the relevant data that were charted that relate to the review questions and objectives. | Table S3 and S5 |
| Synthesis of results | 18 | Summarize and/or present the charting results as they relate to the review questions and objectives. | 5-8 and Table S3 |
| **DISCUSSION** | | | |
| Summary of evidence | 19 | Summarize the main results (including an overview of concepts, themes, and types of evidence available), link to the review questions and objectives, and consider the relevance to key groups. | 8-9 |
| Limitations | 20 | Discuss the limitations of the scoping review process. | 9-10 |
| Conclusions | 21 | Provide a general interpretation of the results with respect to the review questions and objectives, as well as potential implications and/or next steps. | 10-11 |
| **FUNDING** | | | |
| Funding | 22 | Describe sources of funding for the included sources of evidence, as well as sources of funding for the scoping review. Describe the role of the funders of the scoping review. | 11 |

JBI = Joanna Briggs Institute; PRISMA-ScR = Preferred Reporting Items for Systematic reviews and Meta-Analyses extension for Scoping Reviews.

* Where *sources of evidence* (see second footnote) are compiled from, such as bibliographic databases, social media platforms, and Web sites.

† A more inclusive/heterogeneous term used to account for the different types of evidence or data sources (e.g., quantitative and/or qualitative research, expert opinion, and policy documents) that may be eligible in a scoping review as opposed to only studies. This is not to be confused with *information sources* (see first footnote).

‡ The frameworks by Arksey and O’Malley (6) and Levac and colleagues (7) and the JBI guidance (4, 5) refer to the process of data extraction in a scoping review as data charting*.*

§ The process of systematically examining research evidence to assess its validity, results, and relevance before using it to inform a decision. This term is used for items 12 and 19 instead of "risk of bias" (which is more applicable to systematic reviews of interventions) to include and acknowledge the various sources of evidence that may be used in a scoping review (e.g., quantitative and/or qualitative research, expert opinion, and policy document).

*From:* Tricco AC, Lillie E, Zarin W, O'Brien KK, Colquhoun H, Levac D, et al. PRISMA Extension for Scoping Reviews (PRISMAScR): Checklist and Explanation. Ann Intern Med. 2018;169:467–473. [doi: 10.7326/M18-0850](http://annals.org/aim/fullarticle/2700389/prisma-extension-scoping-reviews-prisma-scr-checklist-explanation).

**References (Studies cited in Table S3)**

Arcara, G., Burgio, F., Benavides-Varela, S., Toffano, R., Gindri, P., Tonini, E., et al. (2019). Numerical Activities of Daily Living - Financial (NADL-F): A tool for the assessment of financial capacities (‡). *Neuropsychol Rehabil* 29, 1062–1084. doi: [10.1080/09602011.2017.1359188](https://doi.org/10.1080/09602011.2017.1359188)

Bangma, D. F., Koerts, J., Fuermaier, A. B. M., Mette, C., Zimmermann, M., Toussaint, A. K., et al. (2019). Financial decision-making in adults with ADHD. *Neuropsychology* 33, 1065–1077. doi: [10.1037/neu0000571](https://doi.org/10.1037/neu0000571)

Bechara, A., Damasio, A. R., Damasio, H., and Anderson, S. W. (1994). Insensitivity to future consequences following damage to human prefrontal cortex. *Cognition* 50, 7–15. doi: [10.1016/0010-0277(94)90018-3](https://doi.org/10.1016/0010-0277(94)90018-3)

Benzion, U., Rapoport, A., and Yagil, J. (1989). Discount Rates Inferred from Decisions: An Experimental Study. *Management Science* 35, 270–284. doi: [10.1287/mnsc.35.3.270](https://doi.org/10.1287/mnsc.35.3.270)

Boyle, P. A., Yu, L., Schneider, J. A., Wilson, R. S., and Bennett, D. A. (2019). Scam Awareness Related to Incident Alzheimer Dementia and Mild Cognitive Impairment: A Prospective Cohort Study. *Ann Intern Med* 170, 702–709. doi: [10.7326/M18-2711](https://doi.org/10.7326/M18-2711)

Brand, M., Fujiwara, E., Borsutzky, S., Kalbe, E., Kessler, J., and Markowitsch, H. J. (2014). Game of Dice Task. doi: [10.1037/t31313-000](https://doi.org/10.1037/t31313-000)

Bruine De Bruin, W., Parker, A. M., and Fischhoff, B. (2007). Individual differences in adult decision-making competence. *Journal of Personality and Social Psychology* 92, 938–956. doi: [10.1037/0022-3514.92.5.938](https://doi.org/10.1037/0022-3514.92.5.938)

Finucane, M. L., and Gullion, C. M. (2010). Developing a tool for measuring the decision-making competence of older adults. *Psychology and Aging* 25, 271–288. doi: [10.1037/a0019106](https://doi.org/10.1037/a0019106)

Giannouli, V., Stamovlasis, D., and Tsolaki, M. (2018). Exploring the Role of Cognitive Factors in a New Instrument for Elders’ Financial Capacity Assessment. *JAD* 62, 1579–1594. doi: [10.3233/JAD-170812](https://doi.org/10.3233/JAD-170812)

Green, L., Fry, A. F., and Myerson, J. (1994). Discounting of Delayed Rewards: A Life-Span Comparison. *Psychol Sci* 5, 33–36. doi: [10.1111/j.1467-9280.1994.tb00610.x](https://doi.org/10.1111/j.1467-9280.1994.tb00610.x)

Güth, W., Schmittberger, R., and Schwarze, B. (1982). An experimental analysis of ultimatum bargaining. *Journal of Economic Behavior & Organization* 3, 367–388. doi: [10.1016/0167-2681(82)90011-7](https://doi.org/10.1016/0167-2681(82)90011-7)

Hall, L., Moray, J., Gross, E., and Lichtenberg, P. A. (2022). The Relationship Between Financial Decision-Making and Financial Exploitation in Older Black Adults. *J Aging Health* 34, 472–481. doi: [10.1177/08982643221085407](https://doi.org/10.1177/08982643221085407)

Ho, E. H., Ece, B., Novack, M. A., Pila, S., Karpouzian-Rogers, T., Mather, M. A., et al. (2024). Protocol for a multi-domain scoping review to identify measures of decision-making ability in an ageing population. *BMJ Open* 14, e084178. doi: [10.1136/bmjopen-2024-084178](https://doi.org/10.1136/bmjopen-2024-084178)

Kershaw, M. M., and Webber, L. S. (2008). Assessment of Financial Competence. *Psychiatry, Psychology and Law* 15, 40–55. doi: [10.1080/13218710701873965](https://doi.org/10.1080/13218710701873965)

Lawton, M. P., and Brody, E. M. (1969). Assessment of older people: self-maintaining and instrumental activities of daily living. *Gerontologist* 9, 179–186.

Lejuez, C. W., Read, J. P., Kahler, C. W., Richards, J. B., Ramsey, S. E., Stuart, G. L., et al. (2002). Evaluation of a behavioral measure of risk taking: The Balloon Analogue Risk Task (BART). *Journal of Experimental Psychology: Applied* 8, 75–84. doi: [10.1037/1076-898X.8.2.75](https://doi.org/10.1037/1076-898X.8.2.75)

Lichtenberg, P. A., Campbell, R., Hall, L., and Gross, E. Z. (2020). Context Matters: Financial, Psychological, and Relationship Insecurity Around Personal Finance Is Associated With Financial Exploitation. *The Gerontologist* 60, 1040–1049. doi: [10.1093/geront/gnaa020](https://doi.org/10.1093/geront/gnaa020)

Lichtenberg, P. A., Ocepek-Welikson, K., Ficker, L. J., Gross, E., Rahman-Filipiak, A., and Teresi, J. A. (2018). Conceptual and Empirical Approaches to Financial Decision-making by Older Adults: Results from a Financial Decision-making Rating Scale. *Clin Gerontol* 41, 42–65. doi: [10.1080/07317115.2017.1367748](https://doi.org/10.1080/07317115.2017.1367748)

Lichtenberg, P. A., Stoltman, J., Ficker, L. J., Iris, M., and Mast, B. (2016). Lichtenberg Financial Decision Rating Scale. doi: [10.1037/t45620-000](https://doi.org/10.1037/t45620-000)

Marson, D., Triebel, K. L., Gerstenecker, A., Martin, R. C., Edwards, K., Pankratz, V. S., et al. (2016). Financial Capacity Instrument--Short Form. doi: [10.1037/t51916-000](https://doi.org/10.1037/t51916-000)

McClure, S. M., Ericson, K. M., Laibson, D. I., Loewenstein, G., and Cohen, J. D. (2007). Time Discounting for Primary Rewards. *J. Neurosci.* 27, 5796–5804. doi: [10.1523/JNEUROSCI.4246-06.2007](https://doi.org/10.1523/JNEUROSCI.4246-06.2007)

McHugh, L., and Wood, R. L. (2008). Using a temporal discounting paradigm to measure decision-making and impulsivity following traumatic brain injury: A pilot study. *Brain Injury* 22, 715–721. doi: [10.1080/02699050802263027](https://doi.org/10.1080/02699050802263027)

Mimmack, K. J., Sprague, E. H., Amariglio, R. E., Vannini, P., and Marshall, G. A. (2024). Longitudinal Evolution of Financial Capacity and Cerebral Tau and Amyloid Burden in Older Adults with Normal Cognition or Mild Cognitive Impairment. *J Prev Alzheimers Dis* 11, 966–974. doi: [10.14283/jpad.2023.121](https://doi.org/10.14283/jpad.2023.121)

Mueller, E. A., Wood, S. A., Hanoch, Y., Huang, Y., and Reed, C. L. (2020). Older and wiser: age differences in susceptibility to investment fraud: the protective role of emotional intelligence. *J Elder Abuse Negl* 32, 152–172. doi: [10.1080/08946566.2020.1736704](https://doi.org/10.1080/08946566.2020.1736704)

Rooks, B., Anthony, M., Chen, Q., Lin, Y., Baran, T., Zhang, Z., et al. (2020). A generic brain connectome map linked to different types of everyday decision-making in old age. *Brain Struct Funct* 225, 1389–1400. doi: [10.1007/s00429-019-02013-5](https://doi.org/10.1007/s00429-019-02013-5)

Sellitto, M., Ciaramelli, E., and Di Pellegrino, G. (2010). Myopic Discounting of Future Rewards after Medial Orbitofrontal Damage in Humans. *J. Neurosci.* 30, 16429–16436. doi: [10.1523/JNEUROSCI.2516-10.2010](https://doi.org/10.1523/JNEUROSCI.2516-10.2010)

Sousa, L., Baptista, N., Gomes, C., Peixoto, M., Baeta, É., Rocha, J. C., et al. (2022). Assessment of the Financial Capacity in the Context of Normal and Pathological Cognitive Aging. Preliminary Analysis of the Portuguese Version of the Numerical  Activities of Daily- Living- Financial (NADL-F). *Exp Aging Res* 48, 261–273. doi: [10.1080/0361073X.2021.1974269](https://doi.org/10.1080/0361073X.2021.1974269)

Stewart, C. C., Yu, L., Glover, C. M., Mottola, G., Bennett, D. A., Wilson, R. S., et al. (2020). Loneliness Interacts With Cognition in Relation to Healthcare and Financial Decision Making Among Community-Dwelling Older Adults. *The Gerontologist* 60, 1476–1484. doi: [10.1093/geront/gnaa078](https://doi.org/10.1093/geront/gnaa078)

Yu, L., Mottola, G., Barnes, L. L., Han, S. D., Wilson, R. S., Bennett, D. A., et al. (2021). Correlates of Susceptibility to Scams in Community-Dwelling Older Black Adults. *Gerontology* 67, 729–739. doi: [10.1159/000515326](https://doi.org/10.1159/000515326)
